# Recent Advances in Arsenic Research: Significance of Differential Susceptibility and Sustainable Strategies for Mitigation

**DOI:** 10.3389/fpubh.2020.00464

**Published:** 2020-10-08

**Authors:** Tamalika Sanyal, Pritha Bhattacharjee, Somnath Paul, Pritha Bhattacharjee

**Affiliations:** ^1^Department of Zoology, University of Calcutta, Kolkata, India; ^2^Department of Environmental Science, University of Calcutta, Kolkata, India; ^3^Department of Epigenetics and Molecular Carcinogenesis, U.T. MD Anderson Cancer Center, Smithville, TX, United States

**Keywords:** arsenic toxicity, differential susceptibility, arsenic methylation, single nucleotide polymorphism, epigenetic pattern, sustainable remediation

## Abstract

Arsenic contamination in drinking water and associated adverse outcomes are one of the major health issues in more than 50 countries worldwide. The scenario is getting even more detrimental with increasing number of affected people and newer sites reported from all over the world. Apart from drinking water, the presence of arsenic has been found in various other dietary sources. Chronic arsenic toxicity affects multiple physiological systems and may cause malignancies leading to death. Exposed individuals, residing in the same area, developed differential dermatological lesion phenotypes and varied susceptibility toward various other arsenic-induced disease risk, even after consuming equivalent amount of arsenic from the similar source, over the same duration of time. Researches so far indicate that differential susceptibility plays an important role in arsenic-induced disease manifestation. In this comprehensive review, we have identified major population-based studies of the last 20 years, indicating possible causes of differential susceptibility emphasizing arsenic methylation capacity, variation in host genome (single nucleotide polymorphism), and individual epigenetic pattern (DNA methylation, histone modification, and miRNA expression). Holistic multidisciplinary strategies need to be implemented with few sustainable yet cost-effective solutions like alternative water source, treatment of arsenic-contaminated water, new adaptations in irrigation system, simple modifications in cooking strategy, and dietary supplementations to combat this menace. Our review focuses on the present perspectives of arsenic research with special emphasis on the probable causes of differential susceptibility toward chronic arsenic toxicity and sustainable remediation strategies.

## Introduction

**Figure 1:**
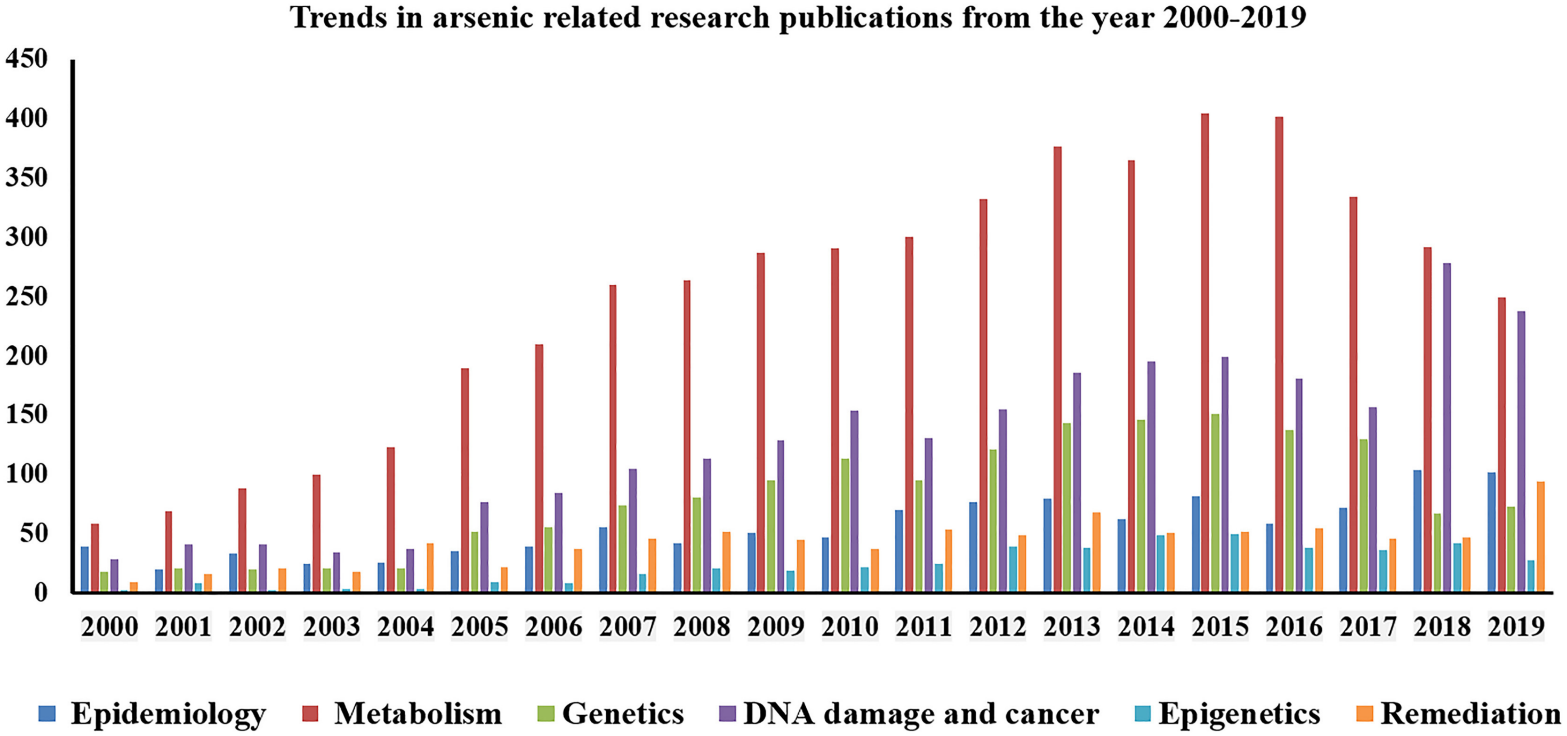
Graphical representation showing trends in research publications on arsenic toxicity (from year 2000 to 2018).

**Figure 2:**
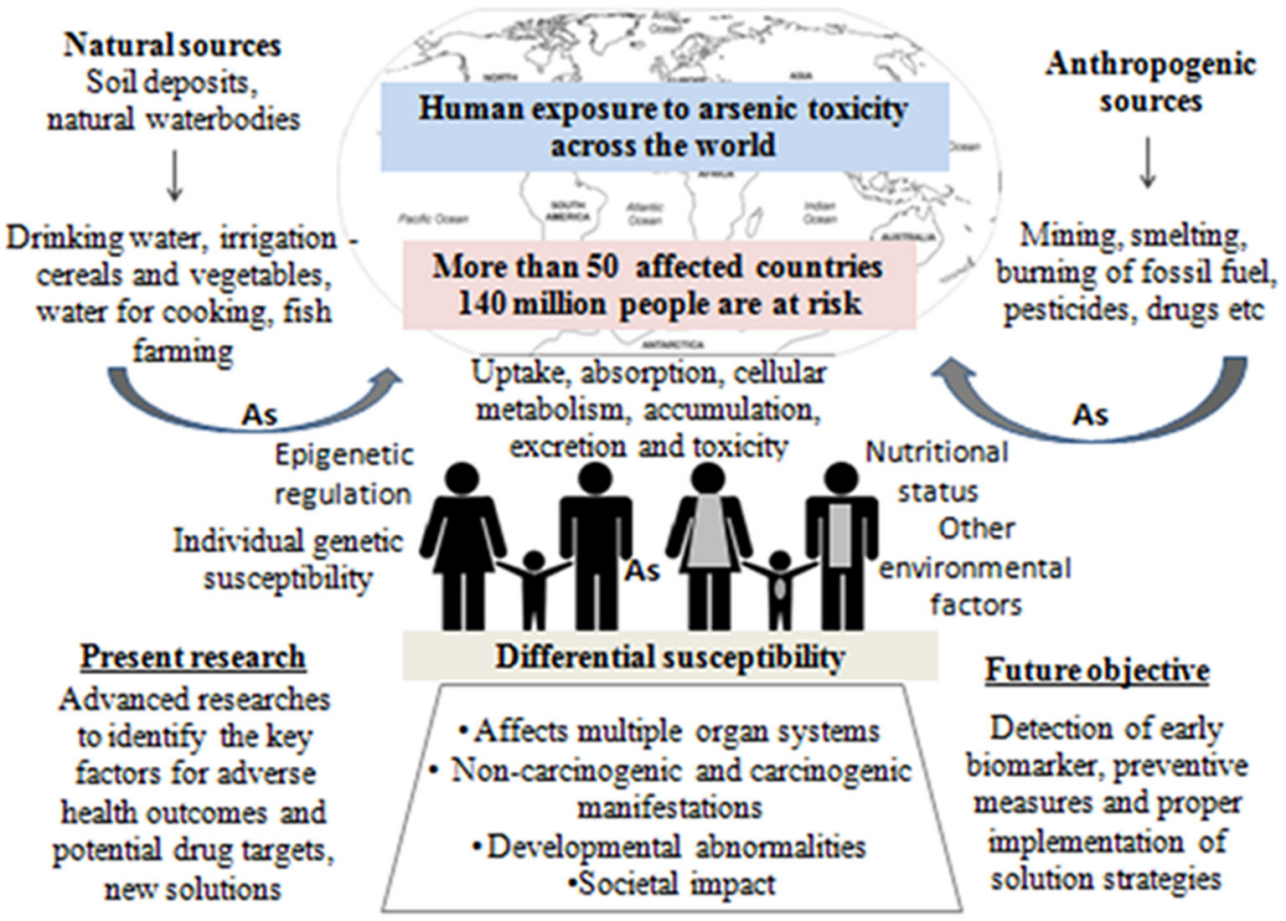
Graphical abstract representing the overall theme of the present review.

Arsenic exposure is one of the major threats to public health in more than 50 nations including China, Australia, India, Bangladesh, Argentina, Brazil, Thailand, Vietnam, Pakistan, Chile, Bulgaria, Canada, Czech Republic, Egypt, Iran, parts of USA, etc. (1). The worldwide scenario of arsenic contamination has been changing with the discovery of newer sites and increasing number of affected people. The latest global count of arsenic-affected individuals, exposed above the WHO safety standard for drinking water of 10 μg/L (1), is ~140 million, which has increased substantially over the decade (2). Since World War II, the initial reports of arsenic toxicity came up-front (3, 4) and have been a prime focus of environmental health research spanning various fields of research including geologists, chemists, pharmacologists, and more so biologists. Arsenic is a metalloid, its inorganic form (e.g., arsenic trioxide, sodium arsenite, and arsenic trichloride are trivalent forms, and lead arsenate and calcium arsenate are pentavalent forms) being found within the natural elements, while organic form circulates within the ecosystem. Common forms of organic arsenic compounds are methylarsonic acid, dimethylarsinic acid, arsanilic acid, etc., formed during metabolism inside living organisms in most of the cases (5). Inorganic arsenic is predominantly found in drinking water and dietary sources like dairy products, meats, cereals, etc. On the other hand, organic form like arsenobetaine is mostly present in seafood, fruits, and vegetables. Arsenic contamination in the groundwater of Indo-Gangetic region occurs from rapid weathering of arsenic-bearing rock in the upper Himalayan catchments, and various river systems get buried in young, low-lying alluvial floodplains of various riverine deltas. The elevated concentration depends on biogeochemical and hydrogeochemical process along with higher sedimentation rate. The slow aquifer-flushing rate is the primary reason for the higher sedimentation in these regions. A potential source of arsenic in the ecosystem is attributed to anthropogenic activities like mining, smelting, and industrial processes, use of arsenic-laden pesticides, etc. (2). Arsenic has three ionized states: arsines As^(III−)^, arsenite As^(III+)^, and arsenate As^(V+)^, the latter two being most mobile in both organic and inorganic forms (2, 5). Both acute and chronic arsenic toxicities generate various deleterious effects in multiple organs and tissues, like hyperkeratosis and change in skin pigmentation, cardiovascular diseases, pulmonary disease, peripheral neuropathy, and developmental and cognitive impairments. Moreover, long-term arsenic exposure even at very low-level causes development of carcinogenic changes in the skin, liver, lung, bladder, and prostate (2). According to recent reports, chronic arsenic exposure around the WHO recommended level (10 μg/L) is also associated with increased risk of urinary tract cancer (6, 7). These evidence indicate that the current guideline for maximum permissible limit of arsenic in drinking water may still present a hazard to the population that are chronically exposed for a long time (8). Earlier researches on arsenic toxicity were primarily focused on population-based epidemiological outcomes, analysis of particular disease risk, chemical, and physiological aspects of arsenic metabolism, study of related gene expression profile, cancer, and DNA damage; all were associated with the mechanism of toxicity and subsequent outcome or disease manifestation. At present, the research perspective shifts toward the study of epigenetic alterations (DNA methylation, histone modification, and miRNA) to justify differential susceptibility toward arsenic exposure, detailed “omics” analysis (whole genome microarray, proteomic, and metabolomic profiling, etc.) of arsenic-induced cancer cases, bioremediation, and development of new therapeutic strategies, which are necessary for combating the outcome of arsenic toxicity as the affected population is increasing around the world. Since the year 2000, with advancement in high throughput techniques, arsenic research has evolved, and newer insights have been discovered (source: PubMed^1^, Figure 1). Research trends from the year 2010 to 2019 indicate that “arsenic metabolism” remained the key focused area for arsenic research (Figure 1). It is indeed important to know the arsenic methylation status, which has been discussed in the following section to understand its toxic effects on biological system, related disease manifestation, and individual susceptibility. The next important focused area was found to be DNA damage and cancer. Understanding epigenetics was increasingly emphasized for the last 5–6 years, where the researchers attempted to explore the role of DNA methylation, histone modification, and miRNA alteration in arsenic toxicity as well as in arsenic-induced carcinogenesis. In this review, we try to string together the recent perspectives of arsenic research with special focus on understanding differential susceptibility in exposed population and how the innovative thinking may soon formulate better remedial strategies against this menace (Figure 2).

## Understanding the Present State of Research

### Sources of Arsenic Exposure

**Figure 3:**
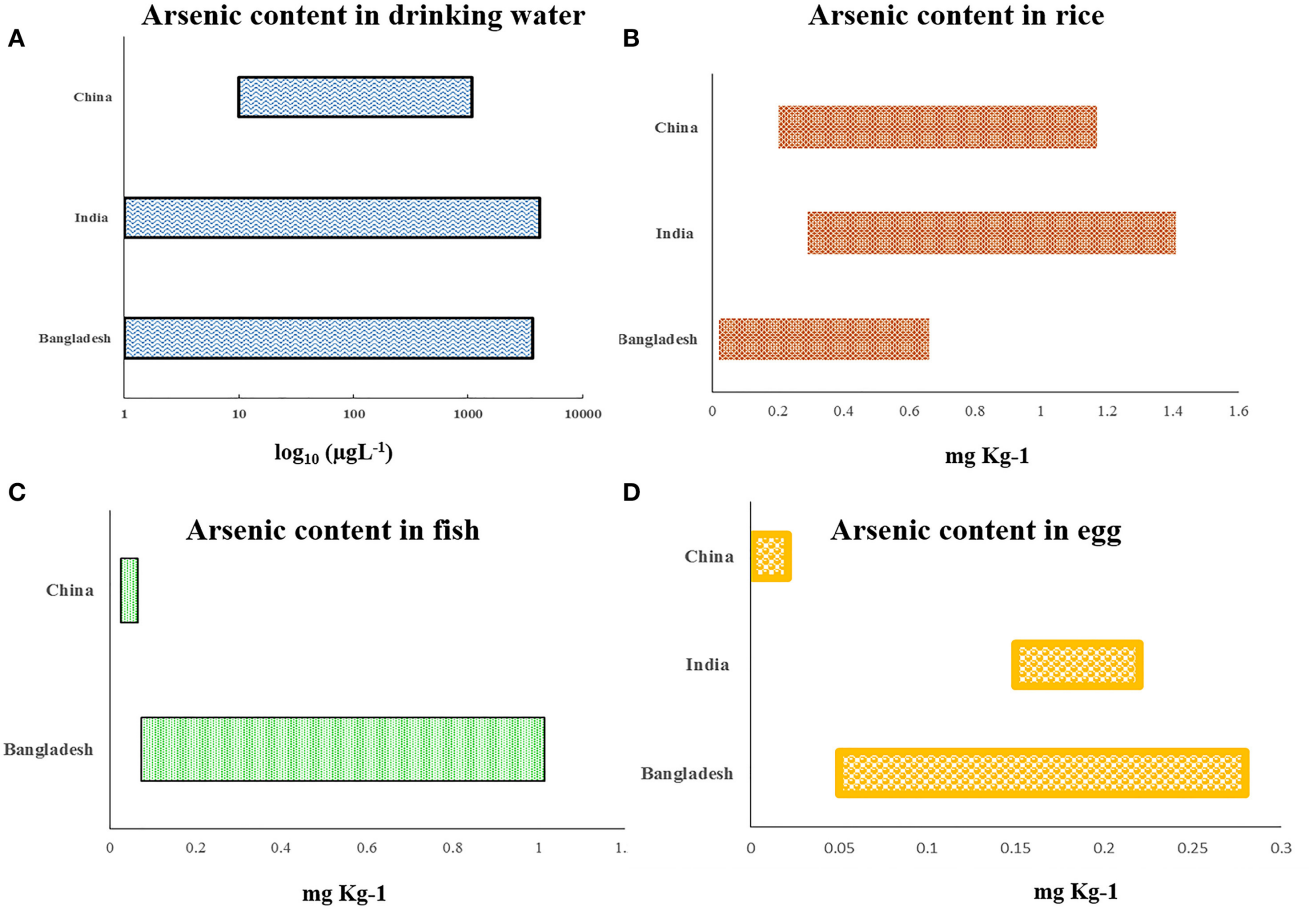
Graphical representation showing recent country-wise reports about arsenic concentration found in **(A)** drinking water, **(B)** rice, **(C)** fish, and **(D)** egg (detailed report is in Supplementary Table 1 with all references).

Arsenic is the 20th most abundant element found in the earth's crust, with an average concentration of 1–2 mg/kg in the continental crust (9). However, there are some geographical hotspots where the content is very high. It includes parts of South America and South and Southeast Asia, the latter two being most populated among all the regions and harboring nearly 75% of the total affected humans mentioned earlier. Arsenic is mobilized into the environment by naturally occurring processes like rainwater leaching, weathering, and seismic and volcanic activities. Another potent source of arsenic is through the emissions of arsenic-laden fumes and wastes that are carried by natural vectors like wind and water, expanding the topological periphery. To date exposure to arsenic is mostly due to groundwater contaminations where inorganic arsenic (iAs) normally exists in the form of arsenite/As^(III+)^ or arsenate/As^(V+)^. Depending on the oxidation potential of the microenvironment, the two states are interconvertible. The pH of the microenvironment regulates the ligand exchange process between the metallic oxides and hydroxides of iAs and the organic intermediates (microbiota) to release arsenic species (10). Higher concentration of arsenic tends to occur in association with metal oxides of iron as well as minerals with high sulfur content. In the Indian subcontinent, the Ganga–Brahmaputra–Meghna basin of the Indo-Bangladesh delta has high deposition of alluvial soil rich in sulfide drained down from the Chota Nagpur region. Increased weathering and rhythmic leaching of arsenic into the water table reflects that the increase in human intervention in abuse of chemical fertilizers laden with arsenic to aid agricultural yield have been associated with incidence of arsenic toxicity (11). Earlier, it was proposed that the organometallic component of arsenic in the groundwater might form complex and subsequently associate strongly with the dissolved arsenic anions, decreasing the release of arsenic, but research has shown that organic decomposition by certain bacteria generates anaerobic conditions whereby they release the arsenic species from these solid phases (12, 13). Apart from drinking water, relevant amount of arsenic exposure has been accounted for diet, especially through rice (Figure 3, Supplementary Table 1). The exposure becomes more pronounced in the region of Bangladesh and India, as the arsenic-affected zone have population whose staple is rice, grown and consumed locally (14). This shows that the dynamic spectrum of arsenic transports into the human physiological domain, the effects of which are being discussed in the following section.

### Arsenic Metabolism: Toxic Nature Chemistry

Majority of the affected population are exposed to arsenic primarily through drinking water and food. Several studies have described the mechanism of arsenic metabolism inside human biological system (15–17). The inorganic form of oxy-anions, including pentavalent arsenite (H_2_${\text{AsO}}_{4}^{-}$) and trivalent arsenate (H_3_AsO_3_) is present abundantly in natural water. Organic form of arsenic is rarely found in the environment (18). The major metabolic pathways of iAs in humans includes several biochemical reactions like oxidation, reduction, methylation, thiolation, and glutathiolation (15, 19), of which methylation is critically important for the toxic pathology, tissue distribution, and cellular retention of arsenic. Arsenic in the form of arsenite/As^(III+)^ or arsenate/As^(V+)^ is absorbed in the gastrointestinal tract (GIT) of human, where arsenite is absorbed more rapidly than arsenate. As^(III+)^ enters into the cell through aquaglyceroporins [AQP3, AQP7, AQP9, and AQP10], and As^(V+)^ uses phosphate transporters, respectively, whereas cellular efflux of arsenic primarily occurs through ATP-binding cassette transporters (MRP1, MRP2, and MRP5). Apart from these, certain glucose transporters (GLUT1 and GLUT5) and organic anion transporting polypeptides were also reported to be responsible for cellular arsenic uptake under different circumstances (15). The initial reduction in arsenate to arsenite is done by liver enzyme arsenate reductase. It is then methylated by arsenic^(III)^ methyl transferase (AS3MT) in the presence of S-adenosyl-methionine (SAM) as methyl donor to monomethylarsonic acid (MMA^v^), which is reduced to monomethylarsonous acid (MMA^III^). MMA^III^ is again methylated to dimethylarsinic acid (DMA^v^), which is subsequently reduced to dimethylarsinous acid (DMA^III^). The main metabolites monomethylarsonic acid (MMA^v^) and dimethylarsinic acid (DMA^v^), where arsenic is in the pentavalent state, are less toxic compared to inorganic arsenic (iAs) and readily excreted in urine. In humans, during metabolism, some arsenic is accumulated (about 40–60%) in skin, hair, nails, muscle, bones, and teeth as iAs and MMA (MMA^III^ and MMA^v^), which may impart toxic effects in multiple tissues and organs in their later life (16, 18, 19). Another pathway of arsenic metabolism shows sequential addition of methyl groups to trivalent arsenicals and its conjugation with glutathione generating intermediate derivatives like arsenic triglutathione (ATG), monomethyl arsenic diglutathione (MADG), and dimethyl arsenic glutathione (DMAG). A third pathway explained about the formation of iAs/protein conjugate and subsequent generation of methylated metabolites (17). Various studies have also shown effect of folate intake and folate metabolism on arsenic metabolism and related disease risks. The amount of folate intake and genetic variants of folate metabolizing enzymes might be responsible for interindividual variation in arsenic metabolism and differential disease susceptibility (19). Relative distribution of arsenic metabolites in urine is commonly used as a biomarker of current exposure and indicates the individualistic metabolism efficiency, which is one of the major causes of differential susceptibility (16, 20). The percentage of urinary metabolite varies between individuals; most of the literature suggests 10–30% iAs, 10–20% MMA, and 60–80% DMA (19, 20). Conventionally, concentration ratios of MMA/iA and DMA/MMA in the urine indicate the methylation capacity of the affected individual. In addition, the activity of AS3MT might have an association with the tissue-specific retention of various arsenic metabolites in the body and subsequently with individual susceptibility (21, 22). Very few reports are available on thio-DMA as urinary metabolite. Raml et al. (23) identified thio-DMA in the urine samples of Bangladeshi women, and also reported from *in vitro* studies that it was about 10-fold more cytotoxic than dimethylarsinate; however, the specific health consequences of such metabolites are not yet known (22). In another work by Taylor et al. (24), the urine samples from arsenic-contaminated seaweed consumers had been analyzed, and thio-DMA was detected as urinary metabolite. Future studies are required to identify and evaluate the possible outcome of such unique metabolites for better understanding of the toxic nature of this metalloid.

### Physiological Manifestations of Arsenic Toxicity

The major hallmark of arsenic toxicity is the occurrence of dermatological lesions of various types like raindrop hypopigmentation, pigmentation, keratosis (palmer and plantar), and even skin cancers like basal cell carcinoma (BCC), squamous cell carcinoma (SCC), and Bowen's disease. Interestingly, only 15–20% of the population show such manifestations (25, 26). Based on the toxic outcome on human health, arsenic is considered as Group I human carcinogen. In recent years, *in vitro* work with human cancer cell lines have helped to unravel various toxic mechanisms related to dermatological health outcomes and cancerous outcomes of the liver, lungs, bladder, and neuronal disorders, but most of the common health outcomes in human to date are dermatological lesions, peripheral neuropathy, liver damage, respiratory disorders, ocular inflammation and irritations, etc. (27–31). In the last decade, arsenic research at the cellular level revealed that arsenic alters the gene expression pattern within the cell and alters telomere length, epigenomic profile, cell cycle, etc. (32–36). One of the most explored aspects of arsenic toxicity in last decade has been on DNA damage and repair mechanism. Studies both in humans as well as cell lines have yielded results, which have made the detection of genetic damage as a reliable biomarker for arsenic-induced toxic outcomes (31, 37–39). Both chromosomal aberrations as well as micronucleus have been associated strongly with arsenic exposure and have shown prominent correlation to arsenic toxicity when compared with unexposed human subjects (40–42).

### New Efforts Toward Understanding Differential Susceptibility

Arsenic-induced characteristic skin lesions have been considered for years as the hallmark of chronic toxicity. We have found that exposed individuals, residing in the same area, show varied dermatological lesion phenotypes even after consuming equivalent amount of arsenic over the same duration of time. In fact, our observation suggests that only a smaller percentage of the exposed individuals show arsenic-induced characteristic skin lesions (43). On the other hand, the precancerous lesions like plantar and palmer hyperkeratosis often lead toward detrimental malignancies in some of the affected individuals, whereas others retain only the precancerous forms lifelong. Different researches indicate several factors like individual arsenic methylation capacity, genetic susceptibility, epigenetic profile, etc. as major role players behind the differential susceptibility (16, 44).

#### Arsenic Methylation Capacity and Differential Susceptibility

The interindividual variation in arsenic methylation potential could be an important predictor of individual's susceptibility. Majority of the researchers found that people with skin lesions and high arsenic exposure are likely to have reduced arsenic methylation capacity with high trivalent species MMA in urine (45–48). The methylation capacity might reduce with increasing dose of arsenic, smoking and alcohol consumption, age, and nutritional folate deficiency (46, 49, 50). On the contrary, one study from China reported about higher methylation capacity among people above 40 years of age compared to below 40 years, but they also found positive correlation between %MMA and risk of skin lesion. On the other hand, women, especially at pregnancy, have increased methylation capacity than men and non-pregnant women, respectively, which may be due to the effect of estrogen, and children also have better methylation capacity than adults (51–53). A case–control study from China reported similar total arsenic concentration in skin lesion and no skin lesion group, but they found increased concentration of MMA among the skin lesion individuals (54), which indicates that efficiency of arsenic methylation is indeed important for differential susceptibility. A detailed account of recent studies regarding arsenic methylation efficiency and risk of skin lesion is summarized in Table 1. Arsenite methyltransferase (As3MT; EC: 2.1.1.137) catalyzes both monomethyl arsenate (MMA) as well as dimethyl arsenate (DMA) using SAM and arsenite and MMA as substrates, respectively. It oxidizes SAM to S-adenosyl L-homocysteine (SAH) in both cases. Recently, using X-ray crystallography, the molecular structure of *As3MT* in conjugation with As^III^ and SAH have been resolved (69). Several single nucleotide polymorphic (SNP) forms of *As3MT* have been identified in relation to arsenic. In the next section, we try to evaluate the structural aspects of *As3MT* polymorphisms along with SNPs in other relevant genes in relation to arsenic-induced toxic outcomes.

**Table 1:** Summary of studies on individual arsenic methylation capacity and risk of skin lesions in chronic arsenic exposed population.

	**Region and sample size**	**Source and analyte**	**Association with primary methylation index (PMI) and arsenic-induced skin lesion individuals (cases)**	**References**
1	Taiwan, 52	Drinking water (urine)	Cases had higher percent of iAs and PMI than matched controls	(55)
2	Central Mexico,104	Drinking water (water and urine)	Cases had higher average MMA concentration compared to no skin lesions	(56)
4	Araihazar, Bangladesh, 1,635	Drinking water (water and urine)	The %MMA in urine and PMI were positively associated in cases, whereas SMI was inversely and % iAs was not associated	(57)
5	Pabna, Bangladesh, 1,200	Drinking water (water and urine)	A 10-fold increase in primary methylation ratio was associated with a 1.50-fold increased risk of skin lesions	(58)
6	China, 327	Drinking water (hair, water, and urine)	The relative proportion of MMA was positively related with skin lesion grade, SMI was negatively related with cases	(59)
7	Matlab, Dhaka, Bangladesh, 504	Drinking water (urine)	Cases had three times higher PMI	(60)
8	Matlab, Dhaka, Bangladesh, 1,030	Drinking water (water and urine)	Higher %MMA was found in cases	(61)
9	South of Shaanxi Province (China), 57	Coal combustion (urine)	Cases had higher urinary arsenic and lower SMI	(51)
10	Inner Mongolia, China, 31	Drinking water (blood)	High PMI and low SMI in cases	(62)
11	Yunnan province, China, 146	Arsenic smelting plant (water, urine)	Cases with increased percentage of MMA	(63)
12	Gansu Province, China, 155	Drinking water (urine)	Increased PMI, and reduced SMI in cases	(54)
13	Huhhot Basin, China, 302	Drinking water (water, urine)	Cases had higher levels of urinary iAs and MMA	(64)
14	Huhhot Basin, China, 302	Drinking water (urinary As)	Cases with higher urinary MMA%	(46)
15	Peoples republic of China, 548	Drinking water (water, urine)	Increased urinary MMA was associated to hyperkeratosis	(65)
16	Araihazar, Bangladesh, 4,794	Drinking water (water and urine)	MMA% was higher in skin lesions and DMA% was higher in without skin lesion group	(52)
17	Inner Mongolia, China, 207	Drinking water (water, urine)	Urinary MMA and iAs concentrations were positively associated with cases	(66)
18	Peoples republic of China, 479	Drinking water (water, urine)	Higher iAs and MMA was associated with cases	(45)
19	Shaanxi province, Inner Mongolia, China, 96	Coal combustion and drinking water (air, water, urine)	Subjects with skin lesions had higher urinary contents of iAs, MMA, and DMA	(67)
20	Pakistan, 398	Drinking water (water and urine)	Higher iAs% and MMA%, lower DMA%, indicating high PMI and low SMI among cases	(48)
21	Araihazar, Bangladesh, 1,464	Drinking water (water and urine)	Decreased urinary %DMA in cases	(68)
22	China, 119	Drinking water (water and urine)	Higher PMI in cases and higher SMI in patient recovery and improvement	(50)

*As, arsenic; iAs, inorganic arsenic; MMA, monomethylated arsenic species; DMA, dimethylated arsenic species; PMI, primary methylation index; SMI, secondary methylation index*.

#### Single Nucleotide Polymorphism and Differential Susceptibility

Previous reports indicate that arsenic-induced health effects might be more deleterious among the exposed population carrying susceptible variants of genes primarily related to arsenic metabolism, oxidative stress, DNA damage repair pathways, etc. Variations in the gene for *AS3MT* have been shown to be the most influential parameter in urinary arsenic metabolites and different disease manifestations including carcinogenic outcome. Several population-based association studies were conducted with a number of SNP sites, among which G>A change in the C10orf32 region (rs 9527) was found to be associated with increased skin lesion risk in Indian (70) and Bangladesh population (71). *AS3MT*, Met287Thr polymorphisms (rs11191439) were reported to be having different arsenic methylation efficiency compared with the wild type and associated with risk of development of skin lesions, bladder cancer, and increased micronucleus frequency (72, 73). Apart from *AS3MT*, other important genes related to arsenic metabolism are Purine nucleoside phosphorylase (*PNP*), methylenetetrahydrofolate reductase (*MTHFR*), methyltetrahydrofolate-homocysteine methyltransferase (*MTR*), cystathionine-beta-synthase (*CBS*), glutathione S-transferase omega 1 (*GSTO1*), and glutathione S-transferase omega 2 (*GSTO2*). *MTHFR* catalyzes the biochemical conversion of 5,10-methylenetetrahydrofolate (5,10-methyl-THF) to 5-methyltetrahydrofolate (5-methyl-THF) during the formation of SAM, which acts as a methyl donor of arsenic methylation. *MTHFR*, C677T, and C1298A polymorphism was reported to be associated with increased urinary MMA%, decreased DMA%, and risk of arsenic-induced skin lesion in arsenic exposed population from Bangladesh, Argentina, and Taiwan (57, 74, 75). Chen et al. (76) observed that individuals with the *MTHFR* 677TT/1298AA and 677CT/1298AA genotypes were 1.66 and 1.77 times more susceptible to develop skin lesions, compared with those having 677CC/1298CC genotype. Catalyzing the reduction in As^V^ to As^III^ is one of the functions of PNP during arsenic metabolism. A case–control study on 428 subjects from arsenic-exposed region of West Bengal, India found that polymorphisms of *PNP*, His20His, Gly51Ser, and Pro57Pro were significantly associated with arsenic-induced skin lesions risk (77). Similar type of studies depicting association between *PNP* SNPs and arsenic-induced health effects were discussed in Supplementary Table 2. *CBS* catalyzes the conversion of homocysteine to cystathionine, which has an influence on arsenic methylation. Two studies on arsenic-exposed population from Argentina found C234709T and G4920037A variants of *CBS* to be associated with urinary MMA% (19, 78). However, to date, there are no reports from any other population regarding the *CBS* polymorphism, and thus, more studies are needed to confirm its association irrespective of ethnicity. *GSTO*s are another group of genes participating in arsenic metabolism. Several studies found significant association of *GSTO1* polymorphic variants with the risk of skin lesion and cancer. Previous studies reported that Ala140Asp was associated with urinary MMA% and skin cancer risk in population chronically exposed to arsenic from drinking water from Bangladesh, Taiwan, and China (54, 57, 74). However, studies on arsenic exposed population from India, Mexico, Hungary, Romania, Slovakia, and USA did not find any significant association of *GSTO1* polymorphism and arsenic-induced disease etiology (72, 77, 79, 80). Luo et al. (81) reported that, for *GSTO2*, AG genotype for rs156697 and the AG genotype or at least one G allele for rs2297235 had an increased risk of arsenic-induced skin lesions, and for *GSTO1*, individuals carrying at least one C allele for the rs11191979 polymorphism or at least one A allele or the AA genotype for rs2164624 or at least one A allele for rs4925 showed a significant risk of arsenic-induced skin lesions. Glutathione S-transferases (GSTs, including GSTM1, GSTT1, and GSTP1) are important protectors for arsenic-related oxidative stress. *GSTP1*, Ile105Val polymorphism was reported to be associated with skin lesion and urinary arsenic profile among arsenic-exposed population from Bangladesh (82), China (83), and Vietnam (22), whereas no association was reported from the population of India (43) and Turkey (84). Ghosh et al. (43) did not find any association of *GSTT1* null genotype with arsenic-induced skin lesion in a study on 422 Indian subjects but reported that GSTM1-positive genotypes are associated with a high risk of skin lesions. On the other hand, McCarty et al. (58) reported that wild-type *GSTT1* is associated with a higher risk of skin lesions than null genotype, but no association was found in case of *GSTM1*. In a recent study on 241 people from Italy, no association was found between arsenic exposure and urinary arsenic profile (85). Genetic variants in BER-pathway-associated genes such as 8-oxoguanine DNA glycosylase (*OGG*), X-ray and repair and cross-complementing groups 1 and 3 (*XRCC1, XRCC3*), and apurinic/apyrimidinic endonuclease (*APE1*) may alter the genotoxicity of arsenic. Multiple case–control studies reported about the association of polymorphic variants of these genes with arsenic-induced disease risk. A detailed description of most recent (considering last 10 years) population-based polymorphism studies stating population size, source of arsenic exposure, and polymorphic variant associated with the particular disease have been summarized in Supplementary Table 2. To date, several population-based studies revealed significant association between the genotypic variants and arsenic-induced disease manifestation; however, the exact mechanistic aspect behind the role of single nucleotide polymorphism of a specific gene in understanding differential susceptibility still remains questionable.

#### Epigenetic Alterations, Gene Expression, and Differential Susceptibility

Recent researches identified epigenetic regulations, which include primarily DNA methylation, histone modification, and miRNA interaction as one of the critical regulators of arsenic-induced disease manifestations. Dynamic reversibility of epigenetic marks is a truly significant property, and it may pave the pathway of epitherapeutics to overcome the road blocks in developing potential drug targets for curing diseases due to arsenic toxicity. Smeester et al. (86) did a comprehensive examination of DNA methylation levels within CpG islands for over 14,000 genes among arsenic exposed with skin lesion (arsenicosis cases) and without skin lesion individuals. They found 182 hypermethylated genes in arsenicosis cases, the majority of which is involved in cancer-associated pathways. A whole genome microarray-based study was conducted on Bangladesh population, where 10 subjects with newly developed skin lesion and 10 no skin lesion were selected from a previous cross-sectional study of 957 individuals to evaluate the possible epigenetic changes. Results indicated DNA methylation changes over time in people having arsenic-induced skin lesions compared to control. They found top 20 differentially methylated CpG sites of which 13 CpGs (*TCEB3B, CYC1, CDH4, RHBDF1, CCDC154, JAKMIP3, AGAP2, PL-5283, CHPF, PPAP2C, PCNT, SLC6A3*, and *MAP3K1*) were increased in % methylation, and 7 CpGs (*MYO3B, KIAA1683, LOC642597, C2orf81, ESRRG, PRDM9*, and *TNXB*) were decreased in % methylation between baseline and follow-up (87). Majumder et al. (88) observed a correlation pattern between different stages of arsenic-induced skin lesion and whole genome DNA methylation. A study on Bangladesh population showed that genomic hypomethylation of peripheral blood lymphocyte DNA is associated with 1.8-fold increase risk for skin lesions (89). Another genome-wide DNA methylation study on 120 individuals from China found changes in global DNA methylation among patients afflicted with arsenical skin lesions. They also depicted about detectable DNA methylation changes due to arsenic exposure over the generations even though exposure occurred decades ago. Chanda et al. (90) observed *GMDS* gene fragment hypermethylation in the peripheral blood leukocyte DNA of skin cancer persons exposed to arsenic and suggested as a biomarker for arsenic-induced cancer. *AS3MT* gene plays an important role in arsenic metabolism and its toxicological response. Gribble et al. (91) found marked promoter hypomethylation *AS3MT* gene in arsenic-exposed population from Arizona, but no reports are found regarding the relationship between skin lesion status and *AS3MT* promoter methylation to date. It will be an interesting and important finding for future researchers, which will help in mechanistic understanding of how epigenetic modification of *AS3MT* contributes in differential susceptibility. In another study by Janasik et al. (92), significant promoter hypermethylation of *NRF2* and *KEAP1* was observed among occupationally arsenic-exposed copper mill workers from Poland. Our group had reported about mitochondrial DNA hypomethylation among arsenic-exposed individuals from highly arsenic affected areas of West Bengal, India, but no significant difference between with and without skin lesion group was observed. However, mitochondrial DNA copy number was found to be significantly elevated among skin lesion individuals with increased expression of electron transport chain complex I, subunit *ND6* and *ND4* genes (93). Recently, we had reported about significant promoter hypomethylation with increased expression of mitochondrial biogenesis regulatory genes, *Tfam* and *PGC1*α, among arsenic-induced skin lesion individuals compared to no skin lesion group, chronically exposed to arsenic through drinking water (94). Few studies also reported about altered promoter methylation of various important genes and subsequent change in gene expression among individuals with skin lesion compared to those without skin lesion explaining the vital role of epigenetic changes behind differential susceptibility (Table 2). High-throughput whole genome omics studies are useful tools to analyze and identify specific gene expression alterations in response to arsenic toxicity. In a study of proteomic profiling of arsenic-induced keratosis samples, three key proteins were identified, which were consistently differentially expressed in lesional skin compared to unaffected skin. The cadherin-like transmembrane glycoprotein, desmoglein 1 (DSG1), was suppressed, whereas the expression of keratin 6c (KRT6C) and fatty acid binding protein 5 (FABP5) were significantly increased (100). Argos et al. (101) analyzed the effect of arsenic toxicity on the development of arsenical skin lesion status by genome-wide gene expression patterns, where the expression of about 22,000 transcripts was evaluated between with skin lesion and without skin lesion group. They found 468 differentially expressed genes between the two groups. The presence of genomic deletion(s) in a number of genes (*OR5J2, GOLGA6L7P, APBA2, GALNTL5, VN1R31P, PHKG1P2, SGCZ, ZNF658*) and long intergenic non-coding RNA (lincRNA) genes (RP11-76I14.1, CTC-535 M15.2, RP11-73B2.2) were associated with higher risk for development of skin lesions independent of gender, age, and arsenic exposure (102). In one study, HaCaT cell line was treated with low dose of arsenic (100 nM sodium arsenite) for 6 months, and then, SILAC-based quantitative proteomics approach resulted in the identification of 2,111 proteins, among which 42 proteins were found to be overexpressed and 54 downregulated upon chronic arsenic exposure (103). Altogether, these studies provide insight into molecular alteration behind differential susceptibility.

**Table 2:** Studies on arsenic induced alterations in promoter DNA methylation related to skin lesion status.

	**Region and sample size**	**Source of arsenic (samples used for estimation)**	**Promoter methylation status of target genes**	**References**
1	West Bengal, India, 158	Drinking water (water)	Hypermethylated promoter region of *p53* and *p21* in skin cancer patients	(95)
2	Murshidabad, West Bengal, India, 122	Drinking water (water, urine)	Significant hypermethylation in the promoters of both *DAPK* and *p16* genes in skin lesion cases compared to no skin lesions	(96)
3	Murshidabad, West Bengal, India, 245	Drinking water (water, urine)	Significant promoter hypomethylation of *ERCC2* gene with increased expression	(97)
4	China, 208	Coal combustion (hair, urine)	Promoter hypermethylation of p15^INK4b^ in arsenical skin lesion group	(64)
5	Guizhou, China, 138	Coal combustion (hair)	Hypermethylation of *ERCC1* and *ERCC2* and suppressed gene expression were found with skin lesion arsenicosis patients	(98)
6	Poland, 111	Copper mill (urine)	Hypermethylation of *NRF2* and *KEAP1* and altered gene expression in occupationally exposed group	(92)
7	Southern Taiwan, 40	Drinking water (water)	Unmethylation at −56 and −54 bp CpG in the CCND1 promoter—a predictor for invasive progression in arsenic induced Bowen's disease patients	(99)
8	Murshidabad, West Bengal, India, 326	Drinking water (water, urine)	Significant promoter hypermethylation of *MLH1* and *MSH2* gene was observed in skin lesion individuals	(38)
9	Murshidabad, West Bengal, India, 390	Drinking water (water, urine)	Promoter hypomethylation and increased gene expression of *Tfam* and *PGC1α* in skin lesion and skin cancer patients	(94)

Another important regulator of epigenetic machinery is the different miRNAs, which may play vital roles behind arsenic-induced individual susceptibility. In a recent study, total miRNA expression analysis was done on premalignant and malignant skin lesion tissues (basal cell carcinoma and squamous cell carcinoma) from an Indian population chronically exposed to arsenic. A total of 35 miRNAs were reported to be differentially expressed among the three lesion types analyzed. Two miRNAs (miR-425-5p and miR-433) were increased in both BCC and SCC relative to hyperkeratosis, indicating their association with malignancy. Two other miRNAs (miR-184 and miR-576-3p) were activated in SCC relative to both BCC and hyperkeratosis, suggesting selective induction in tumors capable of metastasis. Six miRNAs (miR-29c, miR-381, miR-452, miR-487b, miR-494, and miR-590-5p) were selectively suppressed in BCC relative to both SCC and hyperkeratosis (104). A previous study of Banerjee et al. (105) reported about 4.5-fold upregulation of miR21 in skin lesion individuals compared to the no skin lesion group. The expression of the downstream targets of miR21 (PTEN and PDCD4) varied inversely, but the expression of pAKT and PI3K varied proportionately with its expression levels. Another study on arsenic-treated HaCaT cell line identified differential expression of 30 miRNAs of which miR-21, miR-200a, and miR-141 might play a role in skin carcinogenesis (106). Very few studies are so far reported regarding this area, and thus, it is indeed an interesting field for present day researchers to enlighten with newer findings.

Quite a few studies showed arsenic-induced alteration in post-translational histone modifications (PTHM) including H3K36me2, H3K36me3, H3K79me2, H3K27me3, H3K9me2, H3K18ac, H3K9me2, H4K16ac, etc. (107, 108, 108–111)]. Most of them are either *in vitro* or in mouse models, but population-based studies justifying difference between with and without skin lesion individuals are really scarce. Cantone et al. (112) identified that increased H3K4me2 and H3K9ac is associated with inhaled arsenic particulate matter in steel plant workers. Two different studies on arsenic-exposed Bangladesh population observed alteration in H3K9me2, H3K9ac, H3K4me3, H3K27me3, H3K27ac, and H3K36me2 moieties (113, 114). Pournara et al. (115) reported decreased H3K9me3 and unaltered H3K9ac among a population from Argentina, chronically exposed to arsenic through drinking water. A study on Chinese population exposed to arsenic from indoor coal combustion reported that modifications of H3K18ac, H3K9me2, and H3K36me3 are associated with the degree of oxidative damage and the severity of arsenicosis. However, none of the studies particularly differentiate the degree of altered PTHM between arsenic-exposed skin lesion phenotypes compared to those with no skin lesion. Recently, our group has reported about two different PTHMs among chronic arsenic-exposed population from India considering the skin lesion status (38, 116). We identified significant upregulation of H3K79me1 in individuals with arsenic-induced skin lesion, and H3K79me1 was found to be regulated by the upstream methyltransferase DOT1L. Again, significant downregulation of H3K36me3 was found in the arsenic-exposed with skin lesion individuals with an impairment of mismatch repair pathway activated by arsenic-induced DNA damage. Cell-line-based *in vitro* studies and animal model are necessary for the identification of detailed molecular mechanism and for the observation of the outcome of advanced therapeutics (113). However, it is noteworthy to mention that only population-based studies of arsenic toxicity may confer greater opportunities in epitherapeutic drug development and identification of early biomarker for arsenicosis.

## Plausible Mitigation Strategies

One of the ready solutions of arsenic removal is the reduction at source and providing an alternative source of drinking water. Arsenic comes into our body mainly through drinking water sources and a variable dietary intake of food grown in contaminated areas (Figure 3, Supplementary Table 1). With more than 140 million individuals affected, it might not be possible to provide better infrastructure in the removal of arsenic for daily consumption of arsenic-safe diets. The huge economic burden due to the population size makes it difficult to implement the strategies. Though beneficial for the population, the cost of installation of new wells of 1,000 ft deep or more is prohibitive. Another major challenge is the maintenance of already installed filters. After few years of installation, these filters either did not work properly or gets saturated with arsenic.

### Reduction in Arsenic at Source

**Figure 4:**
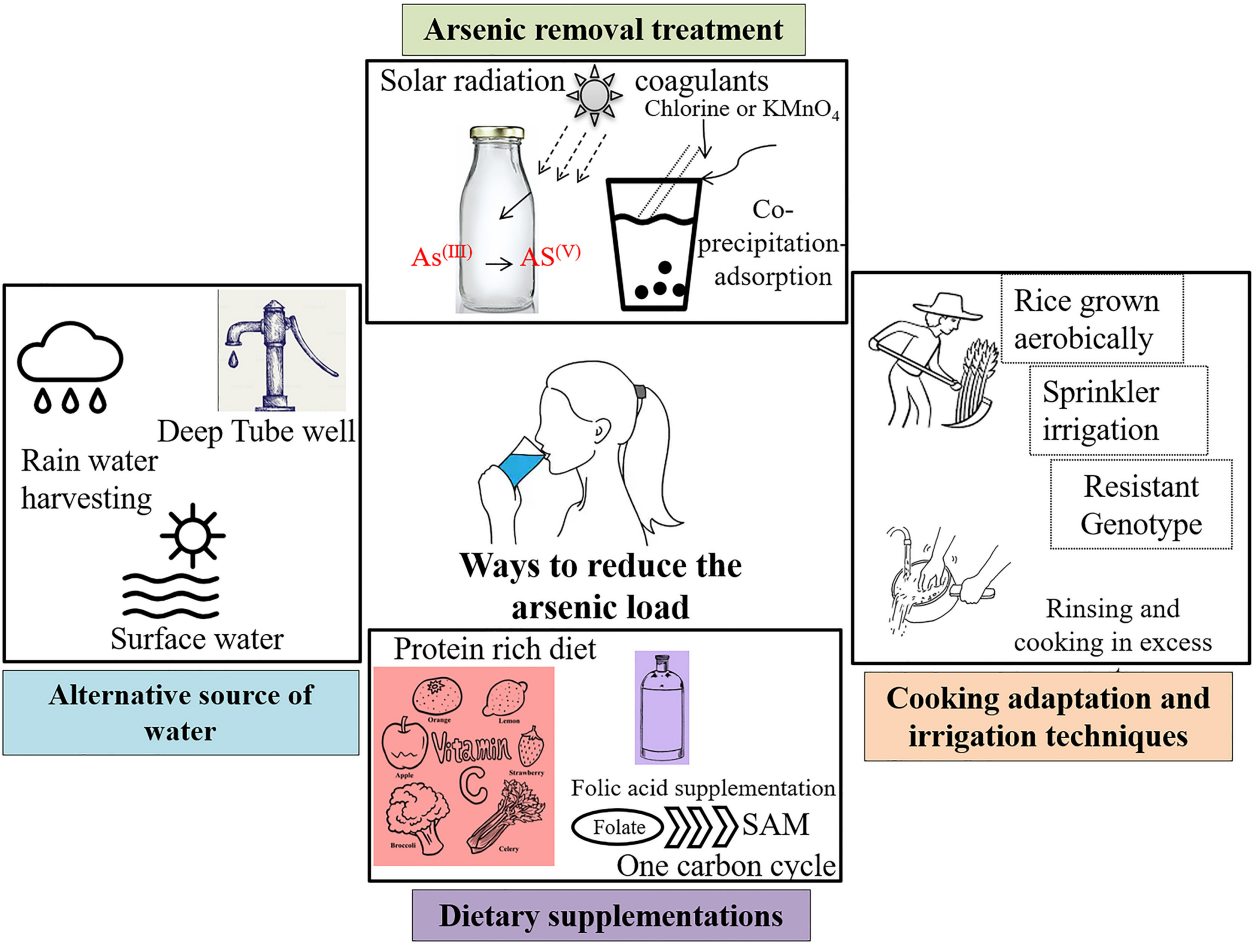
Sustainable solution strategies to combat arsenic toxicity.

This reduction can be done in two ways: (i) alternative arsenic-safe water source and (ii) treatment of arsenic-contaminated water. A comprehensive report is available with detailed pros and cons of the several methods (Wilsonweb, Water supply options^2^). Alternative sources include groundwater [deep tube well, shallow shrouded tube well (SST) and very shallow shrouded tube well (VSST), dug well, infiltration galley], surface water (protected ponds, pond sand filters, combined filters, household filters), and rain water harvesting. Apart from these alternative water sources, arsenic can also be removed from water by various treatments. For example, solar oxidation of arsenic kept in transparent bottles effectively reduce the arsenic load by increased oxidation from As^(III+)^ to As^(V+)^. The common rural practice of storing water for longer period of time in clear container reduces the arsenic concentration through sedimentation (Figure 4). Another effective way to remove arsenic is through coprecipitation and adsorption processes after reduction of As^(III+)^ to As^(V+)^ by bleaching powder (chlorine) or potassium permanganate. Aluminum alum, Al_2_(SO_4_)_3_.18H_2_O, ferric chloride, FeCl_3_, and ferric sulfate, Fe_2_(SO_4_)_3._7H_2_O, are some of the commonly used coagulants. This technology can be used in our daily households with some simple adaptations—popularly known as Bucket Treatment Unit (BTU) (Wilsonweb, Water supply options^2^). Additionally, various sorptive media columns with activated alumina, iron-coated sand, granular ferric hydroxide, etc. are commercially available. The most efficient way to remove arsenic is the use of membrane techniques like reverse osmosis, nano filtration, and electrodialysis, but these are highly expensive for mass scale installation.

### Adaptation of Cooking and Irrigation Practices

Rice being the staple food for billions of people, it significantly contributes more arsenic than any other food source (16, 117). Irrigation with arsenic-contaminated groundwater poses another major threat to public health through food-chain contamination. Conventional irrigation system in flooded paddies increases the arsenic uptake further (117). Recent studies have ascertained that the adaptation of different agronomic techniques can effectively reduce bioaccumulation in rice grains (118). For example, rice grown aerobically significantly has low arsenic accumulation compared to those grown in flooded condition (119). Again, another study reported the reduction in the total As during Sprinkler irrigation compared to traditional flooding (120). Few adaptations in cooking method can effectively lessen levels of arsenic within the grains. For example, a significant decrease was observed by thoroughly rinsing and then cooking the grains in an excessive amount of water, thereby increasing the percolation rate as arsenic is “mobile” in liquid water (117). A study in West Bengal showed that the traditional cooking method is most effective in lowering the concentration and the atab rice type with the lowest arsenic content (121). A recent review has discussed the possibility of genetically modified rice that would accumulate less arsenic within the rice grains (122). Very recently, rice varieties have been screened and developed that accumulate lesser arsenic, as consumption of arsenic-containing rice has been reported to cause genetic damage in humans (123–126). A recent review has discussed the possibility of genetically modified rice that would accumulate less arsenic within the rice grains (122).

### Effects of Microbial Interactions

Microbiological interactions are crucial for the mobilization of arsenic into the aqueous phase leading to arsenicosis (127). Several arsenate-resistant microbes (ARMs) reduce As^(V)^ to As^(III)^ to thrive on the extreme conditions with high arsenic exposure. Recently, scientists have isolated a microorganism, which uses arsenic to “breathe,” from Searles Lake with extremely unfavorable conditions (10 times saltier and 70 times more caustic, alkaline, pH 9.8) and loaded with toxic arsenic (~300 mg/L total arsenic) (Toxic Substances Hydrology Program, The U.S. Geological Survey^3^). These organisms could be used as a way of arsenic removal from water. Later, the applicability of the study remains undetermined (128). Water-usage switch from surface to groundwater to reduce the risk of microbial contamination increased the risk of arsenic contamination. Two rod-shaped Gram-positive bacteria was reported to remove 51.45 and 51.99% of arsenite and 53.29 and 50.37% of arsenate, respectively, from arsenic-containing culture media isolated from Purbasthali block of Burdwan, West Bengal, India (129).

### Dietary Supplementations

Studies have found that administering dietary supplements like vitamin C, different spectra of medicinal plants, drugs, etc. have reduced the effects of arsenic toxicity, although such observations have not been reported or worked out in humans (130–132). Vitamin C or ascorbic acid is a good antioxidant, which explains the reduction in oxidative stress created by arsenic-induced generation of reactive oxygen species (ROS) and in turn ameliorates the ill effects of arsenic within the body. Activation of antioxidative defense system within the body may be better and quicker. Nrf2 pathway is one of the prime pathways for the activation of antioxidant enzymes in the body (133–135). Research has shown that tea polyphenol brings about systemic activation of the Nrf2 pathway (136–138). We discussed in earlier sections that arsenic exposure depletes the methylation pool within the system. Studies suggest that administration of SAM reverses and/or reduces the degree of arsenic-induced DNA damage and anomalies *in vitro* (126, 139). Hence, a concoction of dietary supplements and reduction in arsenic intake can lead to better lives of the population affected by arsenic (Figure 4).

## Discussion

Interindividual susceptibility plays a key role in arsenic toxicity, which is already evident from the multiple research outcomes. Other than the variation in arsenic biotransformation, host-specific genetic make-up, and epigenetic regulation, an array of other environmental and physiological factors including nutritional status, lifestyle, effect of any other inherited or sporadic disease stage, and coexposure toward other heavy metals may regulate the extent of arsenic-induced skin lesion. Differential rate of arsenic methylation has been reported in many studies, where primarily a correlative outcome suggests that a higher primary methylation index is related to risk of skin lesion (*Reduction in Arsenic at Source*, Table 1). However, the complex mechanism of biotransformation behind skin lesion manifestation still remains elusive. Association studies on SNP and arsenic exposure in different populations have identified risk variants for developing skin lesion, several types of cancer, and other adverse diseases (*Adaptation of Cooking and Irrigation Practices*, Supplementary Table 2). However, there is inconsistency in the results because of population from different geographic areas, ethnicity, source of exposure, sample size, etc. Some studies are also limited on a single population and thus needed further validation [Supplementary Table 2, e.g., arsenic-metabolism-related genes *PEMT* and *DHFR* SNPs were validated only in Bangladesh population (140); similarly *CBS* SNP was analyzed only in Argentina population by (19, 78); in case of *DNMTs, Adiponectin*, and *INPP5A* genes single reports were found to date by Seow et al. (140–142), respectively]. The role of epigenetic modification to explain differential susceptibility is one of the most recent advances in arsenic research, having an interesting future prospect in the development of epitherapeutics. Besides the arsenic-related health effects, there are several other problems among the exposed population living in the arsenic-affected countries like India and Bangladesh, such as socioeconomic status, orthodox beliefs and stigma, lack of awareness, and poor maintenance of infrastructure. One of the best ways to counter arsenic-induced health problems is the intake of a protein-rich diet, as study showed that low intake of protein, choline, or methionine can reduce arsenic metabolism and excretion through urine. With a very low income, such a diet is not possible by the majority of the exposed population, leading to an ever-increasing number of susceptible individuals. It is suggested that citrus-based fruits with loads of ascorbic acid can also help in countering such a toxic effect (143, 144). Furthermore, different social stigma, superstitions, and prejudices about the skin symptoms further worsen the situation. In our own studies (38, 93, 116), while performing random sampling in such affected areas, we have faced discontent and discomfort among individuals and family members to acknowledge the fact of skin lesions like skin darkening, black spots, nodules, gangrene, and cancer in limbs, which causes social isolation of the victims. This stigma makes it difficult for our onboard dermatologists to determine whether an individual's dermatological lesion is due to arsenic or something else. While the public health communities and individual research organizations attempted to reach these populations, they refuse to interact with them, as no direct benefit could be offered toward them. Though years of research have been done, clinical implementation remained unsuccessful due to lack of risk–benefit analysis. Large population size is another major hindrance to implement any preventive measure like potable water supply to population at risk. Till now, no comprehensive therapeutic strategy has been tested. The natural means includes reduction in arsenic load in the drinking water, consuming protein-rich diet; ascorbates and polyphenols contained in regular beverages like tea seem to be the likely source of relief. In many parts of India and Bangladesh, the rural population fails to avail of arsenic-free water or high-protein diet due to lack of knowledge, financial restraints, and poor government infrastructure, and, on top of that, social taboo has kept the situation worse. Governments of developing countries cannot support the huge financial burden to set up arsenic-free deep tube wells or to distribute the arsenic removal domestic filter at individual scale. Even though, in few areas, arsenic filtration units have been installed, the durability of these units is less due to lack of filter replacement, poor monitoring, and delayed implementation. Therefore, the combating strategy is preferred to be bottom–up approach, where people will be aware of alternative and safe water sources along with suggested modification in cooking and irrigation practice (Figure 1). A comprehensive plan of action needs to be implemented, which should be disseminated among the affected people through awareness sessions.

## Conclusion

The problem of arsenic poising is intertwined with several other factors like social beliefs, geological risk factors, poverty, and lack of awareness apart from its associated health risks that further worsen the scenario. Arsenic-induced dermatological anomalies often causes social isolation of affected individuals due to beliefs that arsenic poisoning is a “curse” or may be “contagious” in nature. Researches across the globe are trying to develop a sustainable solution, which is still missing. A holistic multidisciplinary approach is needed to combat with the menace. A lot of data have been generated, and several aspects have been explored. Research is now focused more on mechanistic detailing of individual risk of toxicity and developing strategies to counter arsenic toxicity both at the molecular and environmental perspectives. It needs a conjoint/consensus and a multidisciplinary approach to counter this environmental menace, a slow killer and showing an ever-increasing presence over the years.

## Author Contributions

TS had written the manuscript, composed figures, and tables. PB (2nd author) contributed in manuscript writing and figure composition. SP had contributed in manuscript writing. PB (4th author) had supervised the work and given necessary inputs. All authors contributed to the article and approved the submitted version.

## Conflict of Interest

The authors declare that the research was conducted in the absence of any commercial or financial relationships that could be construed as a potential conflict of interest.

## Footnotes

^1^https://www.ncbi.nlm.nih.gov/pubmed/ (accessed February 20, 2019).

^2^http://wilsonweb.physics.harvard.edu/arsenic/conferences/Feroze_Ahmed/Sec_3.htm (accessed February 20, 2019).

^3^https://toxics.usgs.gov/ (accessed February 20, 2019).

## Abbreviations

As: arsenic
DMA^III^: dimethylarsonous acid
DMA^v^: dimethylarsonic acid
DNA: deoxyribonucleic acid
iAs: inorganic arsenic
miRNA: microRNA
MMA^III^: monomethylarsonous acid
MMA^v^: monomethylarsonic acid
RNA: ribonucleic acid
SAM: S-adenosyl-methionine
WHO: World Health Organization.
