# Supplementary material for: Recent Advances in Arsenic Research: Significance of Differential Susceptibility and Sustainable Strategies for Mitigation
Source: Front Public Health. 2020 Oct 8;8:464. doi: 10.3389/fpubh.2020.00464 (PMC7578365; doi:10.3389/fpubh.2020.00464)
Supplement: Supplementary file 1 [file Table_1.pdf]

Supplementary Table 1. Dietary sources responsible for chronic arsenic toxicity.

	Source	Concentration	country	References
1	**Drinking water** (tube well, well water, ground water, aquifers) **(μgL^{-1})**	4 to 5300	Argentina	McClintock et al., 2012
		16.0-73	Australia	Hinwood et al., 2003
		1 to 3644	Bangladesh	Rahman et al., 2006
		10- 350	Brazil	McClintock et al., 2012
		90-860	Chile	Tapia et al., 2018
		10- 1081.25	China	Rodríguez-Lado, 2013
		1-4200	India	Bhattacharya et al., 2011, 2013
		1-45.9	Italy	Achene et al., 2010
		10-2580	Pakistan	shahid et al., 2018
		1 - 632	Vietnam	Agusa et al., 2014
2	**Cereals and pulses (mg Kg^{-1})**			
	• Rice	0.05-0.42	Australia	Tinggi et al., 2014
		0.021–0.66	Bangladesh	Islam et al., 2014
		0.2-1.17	China	Wu et al., 2011
		0.291–1.411	India	Rahaman et al., 2013
	• Maize	0.20–0.55	Bangladesh	Islam et al., 2014
		0.123–0.342	India	Rahaman et al., 2013
	• Wheat	0.027–0.50	Bangladesh	Islam et al., 2014
		0.097–0.211	India	Rahaman et al., 2013
	• Lentil	0.029–0.121	India	Rahaman et al., 2013
	• Gram	0.098–0.213	India	Rahaman et al., 2013
3	**Vegetables, Fruits (mg Kg^{-1})**			
	• Potato	0.25-0.34	Bangladesh	Islam et al., 2014
		0.312–1.464	India	Rahaman et al., 2013
		0.003–0.015	China	Jiang et al., 2016
	• Carrot	0.25-0.34	Bangladesh	Islam et al., 2014
		0.50-0.56	Chile	Pizarro et al., 2016
	• Banana	0.086–0.57	Bangladesh	Islam et al., 2014
		0.10–0.25	Bangladesh	Islam et al., 2014
	• Mango	BDL-0.194	India	Rahaman et al., 2013

4	**Mushroom (mg Kg^{-1})**	≥ 0.4<br>0.27	USA<br>Spain	Seyfferth et al., 2016<br>Melgar et al., 2014
5	**Egg (mg Kg^{-1})**	0.05–0.28<br>0.001–0.020<br>0.155-0.222	Bangladesh<br>China<br>India	Islam et al., 2014<br>Jiang et al., 2016<br>Rana et al., 2012
6	**Meat (mg Kg^{-1})**			
	• Beef	0.008–0.066	Bangladesh	Islam et al., 2014
	• Chicken	0.012–0.074	Bangladesh	Islam et al., 2014
	• Duck	0.015–0.070	Bangladesh	Islam et al., 2014
	• Pork	0.002–0.035	China	Jiang et al., 2016
7	**Fish, shellfish, sea-food (mg Kg^{-1})**			
	• Fish	0.04-0.94<br>0.025–0.074	Bangladesh<br>China	Islam et al., 2014<br>Jiang et al., 2016
	• Marine fish	0.01–0.63	India	Deshpande et al., 2009
	• Shrimp	0.194–0.482	China	Jiang et al., 2016
	• Crab	0.393–0.657	China	Jiang et al., 2016
8	**Cow milk (mg Kg^{-1})**	0.004–0.16	Bangladesh	Islam et al., 2014

References

Achene, L., Ferretti, E., Lucentini, L., Pettine, P., Veschetti, E., Ottaviani, M., 2010.Arsenic content in drinking-water supplies of an important volcanic aquifer in central Italy. Toxicological & Environmental Chemistry. 92, 509-520.

Agusa, T., Trang, P., T., Lan, V., M., Anh, D., H., Tanabe, S., Viet, P., H., Berg, M., 2014. Human exposure to arsenic from drinking water in Vietnam. Sci Total Environ. 488-489, 562-9. doi: 10.1016/j.scitotenv.2013.10.039.

Bhattacharya, P., Mukherjee, A., Mukherjee, A., B., 2011. Arsenic in Groundwater of India. Encyclopedia of Environmental Health. DOI: 10.1016/B978-0-444-52272-6.00345-7

Bhattacharya, P., Mukherjee, A., Mukherjee, A., B., 2013.Groundwater Arsenic in India: Source, Distribution, Effects and Alternate Safe Drinking Water Sources. Reference Module in Earth Systems and Environmental Sciences, Elsevier.

Deshpande, A., Bhendigeri, S., Shirsekar, T., Dhaware, D., Khandekar, R., N., 2009. Analysis of heavy metals in marine fish from Mumbai Docks. Environ Monit Assess. 159, 493–500.

Hinwood, A., L., Sim, M., R., Jolley, D., de Klerk, N., Bastone, E., B., Gerostamoulos, J., Drummer, O., H., 2003.Hair and toenail arsenic concentrations of residents living in areas with high environmental arsenic concentrations. Environ Health Perspect. 111, 187-193.

Huang, C. Y., Lin, Y. C., Shiue, H. S., Chen, W. J., Su, C. T., Pu, Y. S., Hsueh, Y. M., et al., 2018. Comparison of arsenic methylation capacity and polymorphisms of arsenic methylation genes between bladder cancer and upper tract urothelial carcinoma. Toxicology Letters, 295, 64–73.

Huang, C. Y., Su, C. T., Chu, J. S., Huang, S. P., Pu, Y. S., Yang, H. Y., Hsueh, Y. M., 2011. The polymorphisms of P53 codon 72 and MDM2 SNP309 and renal cell carcinoma risk in a low arsenic exposure area. Toxicology and Applied Pharmacology, 257(3), 349–355.

Huang, C.Y., Chung, C.J., Pu, Y., S., Lin, Y., C., Wu, C., C., Shiue, H., S., Huang, Y., K., 2013. Polymorphism of inflammatory genes and arsenic methylation capacity are associated with urothelial carcinoma. Toxicology and Applied Pharmacology, 272(1), 30–36.

Huang, C.Y., Huang, Y.L., Hsueh, Y.M., Lin, Y.C., Chiang, C.I., Chen, W.J., … Shiue, H.S., 2014. XRCC1 Arg194Trp and Arg399Gln polymorphisms and arsenic methylation capacity are associated with urothelial carcinoma. Toxicology and Applied Pharmacology, 279(3), 373–379.

Islam, M., S., Ahmed, M., K., Habibullah-Al-Mamun, M., Islam, K., N., Ibrahim, M., Masunaga, S., 2014.Arsenic and lead in foods: a potential threat to human health in Bangladesh.Food AdditContam Part A Chem Anal Control Expo Risk Assess. 31,1982-92.

Jiang, Y., Chao, S., Liu, J., Yang, Y., Chen, Y., Zhang, A., Cao, H., 2016.Source apportionment and health risk assessment of heavy metals in soil for a township in Jiangsu Province, China.Chemosphere.168, 1658-1668.

McClintock, T., R., Chen, Y., Bundschuh, J., Oliver, J., T., Navoni, J., Olmos, V., Lepori, E., V., Ahsan, H., Parvez, F., 2012.Arsenic exposure in Latin America: biomarkers, risk assessments and related health effects.Sci Total Environ. 429, 76-91.

Melgar, M., J., Alonso, J., García, M., A., 2014. Total contents of arsenic and associated health risks in edible mushrooms, mushroom supplements and growth substrates from Galicia (NW Spain). Food and Chemical Toxicology 73, 44–50.

Pizarro, I., Gómez-Gómez, M., León J., Román D., Antonia Palacios, M.,2016. Bioaccessibility and arsenic speciation in carrots, beets and quinoa from contaminated area of Chile. Science of the Total Environment 565, 557–563.

Rahaman, S., Sinha, A., C., Pati, R., Mukhopadhyay, D., 2013.Arsenic contamination: a potential hazard to the affected areas of West Bengal, India. Environ GeochemHealth. 35,119–132.

Rana, T., Bera, A., K., Mondal, D., K., Das, S., Bhattacharya, D., Samanta, S., Pan, D., Das, S., K., 2014. Arsenic residue in the products and by-products of chicken and ducks: a possible concern of avian health and environmental hazard to the population in West Bengal, India. Toxicol Ind Health. 30(6), 576-80. doi: 10.1177/0748233712462467.

Seyfferth, A., L., McClatchy, C., Paukett, M., 2016.Arsenic, Lead, and Cadmium in U.S. Mushrooms and Substrate in Relation to Dietary Exposure. Environ Sci Technol. 50, 9661-70.

Shahid, M., Niazi, N., K., Dumat, C., Khalid S., Bibi, I., 2018. A meta-analysis of the distribution, sources and health risks of arsenic-contaminated groundwater in Pakistan. Environmental Pollution 242, 307-319.

Tapia, J., S., Valdés, J., Orrego, R., Tchernitchin, A., Dorador, C., Bolados, A., Harrod, C., 2018.Geologic and anthropogenic sources of contamination in settled dust of a historic mining port city in northern Chile: health risk implications.PeerJ.6, e4699.

Tinggi, U., Schoendorfer, N., Scheelings, P., Yang, X., Jurd, S., Robinson, A., Smith, K., &Piispanen, J., 2014. Arsenic in rice and diets of children. Toxicology and Industrial Health 30, 576–580.

Wu, C., Ye, Z., Shu, W., Zhu, Y., Wong, M., 2011.Arsenic accumulation and speciation in rice are affected by root aeration and variation of genotypes.Journal of Experimental Botany, 62, 2889–2898.
