# Supplementary material for: Recent Advances in Arsenic Research: Significance of Differential Susceptibility and Sustainable Strategies for Mitigation
Source: Front Public Health. 2020 Oct 8;8:464. doi: 10.3389/fpubh.2020.00464 (PMC7578365; doi:10.3389/fpubh.2020.00464)
Supplement: Supplementary file 2 [file Table_2.pdf]

Supplementary table 2. Genetic polymorphisms involved in arsenic induced differential susceptibility and risk of disease manifestation (Reports from last 10 years)

Genes involved in arsenic metabolism pathway

AS3MT (Arsenite Methyltransferase)

	Region and sample size	Source of arsenic (samples used for estimation)	Polymorphism	Association	References
1.	Taiwan; 863	Drinking water (water)	Met (287) Thr	For high exposure level (>50 μg/L), the T-C haplotype is associated with a high risk of carotid atherosclerosis.	Hsieh et al., 2011
2.	USA ,492	Drinking water (urine)	Met (287) Thr<br>T>C	One or more copies of the C allele had an elevated risk of bladder cancer.	Beebe-Dimmer et al.,2012
3.	Texas,499	Drinking water (water)	A35991G (rs10748835)	Associated with hyperlipidemia and hypertension	Gong et al.,2012
4.	USA, 2023	Drinking water (water)	rs1046778, rs3740391, rs3740392, rs3740393, rs11191439, rs7085854, rs10748835, rs11191454	No association was found with bladder cancer.	Lesseur et al., 2012
5.	Bangladesh, 2879	Drinking water (urine)	G>A<br>(rs 9527)	A allele was associated with decreased DMA% and increased skin lesion risk	Pierce et al., 2012
6.	Bangladesh, 1742	Drinking water (water, urine)	rs11191439	wild type rs11191439 was significantly associated with lower levels of urinary MMA%.	Rodrigues et al., 2012
7.	Mexico,255	Drinking water (urine)	M287T,<br>G4965C	Associated with higher levels of urinary<br>DMA%, arsenic metabolism and diabetes.	Drobna et al.,2013
8.	Bangladesh,2060	Drinking water (urine)	2 SNPs in the 10q24.32 region	Independent associations with metabolism efficiency skin lesion risk.	Pierce et al.,2013

9.	Texas,526	Drinking water (urine)	A35991G, (rs10748835)	Associated with cognitive functioning.	Edwards et al.,2014
10.	China, 242	Drinking water (urine)	Met (287) Thr	No association was found.	Fu et al., 2014
11.	Chile, 207	Copper mine industry (urine)	Met (287) Thr	Associated with arsenic-induced cytogenetic damage.	Hernandez et al.,2014
12.	Bangladesh, 1078	Drinking water (urine)	rs10883790, rs11191442, rs3740392	Associated with urinary As and carotid intima-media thickness (cIMT).	Wu et al., 2014
13.	Eastern Europe,1062	Drinking water (urine)	rs3740400 A/C, rs3740393 G/C, rs11191439 T/C, rs1046778 T/C	Associated with methylation efficiency and risk of basal cell carcinoma.	Engstrom et al.,2015
14.	India, 315	Drinking water (water, urine)	C10 or f32 region (rs 9527; G>A)	G>A change in the C10orf32 region was associated with increased skin lesion risk.	Das et al., 2016
15.	Bangladesh, 4794	Drinking water (water, urine)	10q24.32/AS3MT region. rs9527, rs11191527	rs9527 and rs11191527 were strongly associated with DMA%, but not with PMI.	Jansen et al.,2015
16.	American Indian tribes, USA	Drinking water (urine)	rs12768205, rs3740394, rs3740393	Genetic variants were associated with As metabolism, MMA and DMA.	Balakrishnan et al.,2017
17.	Taiwan, 1241	Drinking water (urine)	rs3740393, rs3740392, rs11191438, rs3740391, rs11191439, rs11191453, rs11191454, rs10748835, rs1046778	rs11191438, rs10748835, and rs1046778 were related to the risk of bladder cancer and urothelial carcinoma, while rs3740393, rs11191453, and rs11191454 were associated with As methylation capacity.	Huang et al., 2018
18.	China, 850	Drinking water (urine)	(rs1046778, rs10748835, rs10883790, rs11191438, rs11191442, rs11191454, rs12416687, rs3740390, rs3740392, rs3740393, rs7085104, rs7085854, rs7098825	Not associated with skin lesion.	Luo et al., 2018

26.	Bangladesh, 1613	Drinking water (urine)	rs9527, rs1046778, rs1046778	Associated with urinary %DMA in pregnant woman.	Gao et al., 2019
27.	Croatia, 312	Sea food consumption (urine)	rs7085104, rs3740400, rs3740393, rs3740390, rs11191439, rs10748835, rs1046778	Study showed 'protective' effect of six individual SNPs (rs7085104, rs3740400, rs3740393, rs3740390, rs10748835 and rs1046778, minor alleles) and their haplotype in non-pregnant women only (lower MMA% and higher SMI).	Stajnko et al., 2019
***MTHFR* (*Methylenetetrahydrofolate reductase*)**					
1.	Taiwan, 450	Drinking water (water, urine)	C677T	The CT or TT genotype was associated with decreased urinary DMA% and decreased folate levels and increased risk of urothelial carcinoma.	Chung et al., 2010
2.	Southeastern Michigan ,492	Drinking water (urine)	rs1476413	Lower risk of bladder cancer was observed among homozygotes for the A allele in exposed population.	Beebe-Dimmer et al., 2012
3.	Bangladesh, 1464	Drinking water (urine)	C677T (rs1801133)	The T allele of 677 C> T was associated with higher %MMA, and lower %DMA, but not with skin lesions.	Niedzwiecki et al., 2018
4.	Croatia, 312	Sea food consumption (urine)	rs1801131, rs1801133	No association observed.	Stajnko et al., 2019
PNP (purine nucleoside phosphorylase)					
1.	Taiwan; 50	Sea food (urine)	rs1760940, rs17884106, rs1049562, rs1049564, rs1130650	Not associated with urinary arsenic	Hwang et al., 2010
2.	Taiwan; 863	Drinking water (water)	Gly51Ser, Pro57Pro	For high exposure level (>50 μg/L), the A-T haplotype was associated with high risk of carotid atherosclerosis.	Hsieh et al., 2011
3.	Bangladesh, 1078	Drinking water (urine)	rs17886095, rs17882804, rs3790064	Associated with urinary As and cIMT	Wu et al., 2014
4.	China, 850	Drinking water (urine)	rs1713420,rs1760940,rs3790064,	rs3790064 was associated with arsenic-induced skin lesions.	Luo et al., 2018

5.	Taiwan, 1241	Drinking water (urine)	rs1049562, rs1049564, rs1130650;	No association was found.	Huang et al., 2018
PEMT (Phosphatidylethanolamine N-methyltransferase)					
1	Bangladesh; 940	Drinking water (water)	rs2278952	Polymorphism significantly modified arsenic induced skin lesion status.	Seow et al., 2015
DHFR (Dihydrofolate reductase)					
1	Bangladesh; 940	Drinking water (water)	rs1650697	Polymorphism significantly modified arsenic induced skin lesion status.	Seow et al., 2015
CBS (C**ystathionine beta-synthase**)					
1.	Argentina; 142	Drinking water (water, urine)	C234709T, G4920037A	Associated with urinary MMA%.	Porter et al.,2010
2.	Argentina; 120	Drinking water (water, urine)	C234709T, G4920037A	Associated with urinary MMA% and lung cancer.	Steinmaus et al., 2010
MTR (5-Methyltetrahydrofolate-Homocysteine Methyltransferase)					
1.	Argentina; 142	Drinking water (water, urine)	Asp919Gly (rs1805087)	No association was found.	Porter et al., 2010
2.	Mexico, 2044	Drinking water (urine)	2756A > G (rs1805087)	MTR 2756A>G polymorphism might confer protection for Breast Cancer associated with iAs exposure.	Gamboa-Loira et al., 2017
HO-1 (Heme oxygenase 1)					
1.	Taiwan; 787	Drinking water (water)	Short GT-repeat HO-1	Short GT-repeat HO-1 short allele was associated with a low risk of carotid atherosclerosis.	Wu et al., 2010
2.	Taiwan; 894	Drinking water (water)	Short GT-repeat HO-1	short allele was associated with a low risk of cardiovascular mortality in people with hypertension.	Wu et al., 2011b
3.	Taiwan; 280	Drinking water (urine)	Short GT-repeat HO-1	No association with skin cancer.	Hsu et al., 2015
4.	Taiwan; 1,013	Drinking water (water)	Short GT-repeat HO-1	participants with the Short/Short genotype had an increased risk of Bowen's disease invasive skin cancer and lung squamous cell carcinoma versus those with Long/Short or Long/Long genotype.	Wu et al., 2016

Gene involved in arsenic transportation pathway

ABCC1/ MRP1(Multidrug resistance-associated protein 1)

1.	Turkey; 95	Smelting work (urine, blood)	G1666A	Polymorphism was associated with urinary As concentration.	Kaya et al., 2016

Genes involved in oxidative stress response pathway

GSTO1 (Glutathione S-transferase omega 1)

1.	Taiwan; 400	Drinking water (urine)	Ala140Asp, Glu208Lys	Wild-type Ala140Asp was associated with high urinary MMA%.	Chung wt al., 2011
2.	Taiwan; 863	Drinking water (water)	Ala140Asp,	For high exposure (>50 μg/L), haplotypes were associated with a high risk of carotid atherosclerosis.	Hsieh et al., 2011
3.	Taiwan; 764	Drinking water (urine)	rs4925 (Ala140Asp), rs11509438(Glu208Lys), rs15032 (Thr217Asn)	Ala140Asp was associated with increased urothelial cancer risk.	Hsu et al., 2011
4.	Michigan, USA, 492	Drinking water (urine)	GSTO-1 (rs4925, rs2282326)	No association was found.	Beebe-Dimmer et al., 2012
5.	Taiwan; 247	Drinking water (urine)	Ala140Asp	Asp allele was associated with low urinary MMA% and a slightly high risk of metabolic syndrome	Chen et al., 2012
6.	Bangladesh, 1742	Drinking water (water, urine)	Ala140Asp, rs4925	Homozygous wild type was significantly associated with higher MMA and DMA in urine.	Rodrigues et al., 2012
7.	China, 242	Drinking water (urine)	Ala140Asp, Thr217Asn, Ala236Val	The %DMA of individuals with the Ala/Asp genotype was reduced significantly compared with the Ala/Ala genotype.	Fu et al., 2014
8.	Nadia, West Bengal, India, 38	Drinking water and food, (urine, hair, nail)	Ala 140 Asp	Association of GSTO1 A140D polymorphism and global DNA methylation in the arsenic affected population	Majumdar et al., 2017
9.	China, 850	Drinking water (urine)	rs11191979, rs2164624, rs2282326 and rs4925	Individuals carrying at least one C allele for the rs11191979 polymorphism, at least one A allele or the AA genotype for rs2164624 or at least one A allele for rs4925 showed a significant risk of arsenic-induced skin lesions	Luo et al., 2018

GSTO2 (Glutathione S-transferase omega 1)					
1.	Taiwan; 400	Drinking water (urine)	Asn142Asp	Associated with urothelial carcinom risk.	Chung et al., 2011
2.	Taiwan; 863	Drinking water (water)	Asn142Asp, A183G	For high exposure (>50 µg/L), haplotypes were associated with a high risk of carotid atherosclerosis	Hsieh et al., 2011
3.	Taiwan; 764	Drinking water (urine)	rs2297235 Exon2 5'UTR (-183) rs156697 Asn142Asp	Associated with an increased urothelial cancer risk when compared to the all-wildtype reference, respectively.	Hsu et al., 2011
4.	USA, 2023	Drinking water (water)	Asn142Asp	Polymorphism was associated with bladder cancer risk	Lesseur et al., 2012
5.	China, 242	Drinking water (urine)	GSTO2 Asn142Asp	No association was found.	Fu et al., 2014
7.	China, 850	Drinking water (urine)	GSTO2 SNPs rs156697 and rs2297235	Subjects who carried the AG genotype for rs156697 and the AG genotype or at least one G allele for rs2297235 had an increased risk of arsenic-induced skin lesions.	Luo et al., 2018
GSTP1 (Glutathione S-transferase Pi 1)					
1.	Vietnam; 100	Drinking water (hair, water, urine)	Ile105Val	polymorphisms were associated with the urinary As profile.	Agusa et al., 2010
2.	Taiwan; 764	Drinking water (urine)	Ile105Val	No association with urothelial carcinoma.	Hsu et al., 2011
3.	USA, 2023	Drinking water (water)	Ile105Val	Polymorphism was associated with bladder cancer risk	Lesseur et al., 2012
4.	Taiwan; 280	Drinking water (urine)	Ile105Val	No association with skin cancer.	Hsu et al., 2015
5.	Turkey, 95	Smelting work (urine, blood)	Ile105Val	No association with the blood or urinary As levels.	Kaya et al., 2016
6.	China, 398	Drinking water (hair)	Ile105Val Rs 1695	No association was found with hypertension.	Yu et al., 2017

GSTT1 (Glutathione S-transferase theta 1)					
1.	Vietnam; 100	Drinking water (hair, water, urine)	null genotype of GSTT1	No association with the urinary As profile.	Agusa et al., 2010
2.	USA, 2023	Drinking water (water)	null genotype of GSTT1	Greater risk of bladder cancer in the high arsenic exposure group associated with GSTT1 null.	Lesseur et al., 2012
3.	Taiwan; 280	Drinking water (urine)	null genotype of GSTT1	Null genotype of GSTT1 is associated with skin cancer.	Hsu et al., 2015
4.	China, 230	Drinking water (urine)	null genotype of GSTT1	Associated with higher urinary secondary methylation index.	Yang et al., 2015
5.	Italy,241	Drinking water (urine)	null genotype of GSTT1	significant associations between GSTT1 and the As concentration and metabolite patterns in the urine.	Borghini et al., 2016
***GSTM1* (*Glutathione S-transferase mu 1*)**					
1.	Vietnam; 100	Drinking water (hair, water, urine)	null genotype of GSTM1	Null genotype of GSTM1 is associated with low DMA%.	Agusa et al., 2010
2.	USA, 2023	Drinking water (water)	null genotype of GSTM1	Greater risk of bladder cancer associated with null genotype	Lesseur et al., 2012
3.	Taiwan; 280	Drinking water (urine)	null genotype of GSTM1	No association with skin cancer.	Hsu et al., 2015
4.	China, 230	Drinking water (urine)	null genotype of GST1	Associated with urinary As methylation.	Yang et al., 2015
5.	Italy,241	Drinking water (urine)	null genotype of GSTT1	No association was found.	Borghini et al., 2016

**GSTZ1 (*Glutathione S-transferase zeta 1*)**					
1.	USA, 2023	Drinking water (water)	Glu32Lys	Polymorphism was associated with As induced bladder cancer risk.	Lesseur et al., 2012
MnSOD (manganese-dependent superoxide dismutase)					
1.	Taiwan; 240	Drinking water (urine)	Ala9Val	High urinary As level, 9Val/Ala and 9Ala/Ala polymorphisms were associated with a high risk of hypertension.	Chen et al., 2012

EPHX1 (Epoxide hydrolase 1)					
1.	Taiwan; 280	Drinking water (urine)	Tyr113His, His139Arg	Polymorphisms of Tyr113His were associated with skin cancer.	Hsu et al., 2015
2.	China, 398	Drinking water (hair)	rs1051740 and rs2234922	No association was found with hypertension.	Yu et al., 2017
**Genes involved in DNA damage repair pathway and tumour suppressor genes**					
ERCC2/ XPD (Excision Repair Cross-Complementing Rodent Repair Deficiency, Complementation Group 2/ Xeroderma Pigmentosum Group D)					
1.	USA, 891	Drinking water (water, nail)	A751C (rs171140) G312A(rs1799793)	G312A polymorphism was associated with bladder cancer risk	Andrew et al., 2010
2.	China; 486	Indoor coal combustion (hair, urine)	G23591A, A35931C	Polymorphisms of A35931C were potentially associated with skin lesions.	Lin et al., 2010
3.	Taiwan; 981	Drinking water (urine)	Lys751Gln	No association with urothelial carcinoma and urinary As profiles.	Chiang et al. 2014
4.	Taiwan; 280	Drinking water (urine)	C156A, rs238406	Associated with arsenic induced skin lesion.	Hsu et al., 2015
5.	Taiwan; 540	Drinking water (urine)	Lys751Gln	No association with renal cell carcinoma	Hsueh et al, 2017

XRCC1 (X-ray repair cross-complementing protein 1)					
1.	Taiwan; 981	Drinking water (urine)	Arg194Trp, Arg399Gln	399Gln/Gln and 194Arg/Trp and 194Trp/Trp were associated with urothelial carcinoma.	Chiang et al., 2014
2.	Taiwan; 280	Drinking water (urine)	Arg280His, Arg399Gln	No association with skin cancer.	Hsu et al., 2015
3.	Italy,241	Drinking water (urine)	Arg399Gln	Interaction between Arg399Gln and arsenic exposure was observed in connection with telomere length.	Borghini et al., 2016

4.	Vietnam, 100	Drinking water (urine)	Arg194Trp, Arg280His, Pro206Pro, and Arg399Gln	Both Arg/Arg homozygous subjects and Arg/Gln heterozygous individuals showed a significantly higher urinary inorganic As percentage.	Fujihara et al., 2016
5.	Taiwan; 540	Drinking water (urine)	Arg194Trp, Arg399Gln	194Trp correlates with a decreased risk of RCC in a human population with low exposure to arsenic.	Hsueh et al, 2017
XRCC3 (X-ray repair cross-complementing protein 3)					
1.	USA, 891	Drinking water (water, nail)	Thr241Met	Elevated bladder cancer risk was associated with toenail arsenic levels in heterozygotes compared to wildtype	Andrew et al., 2010
2.	West Bengal, India; 421	Drinking water (water, urine)	Thr241Met	Met allele was associated with a low risk of skin lesions, peripheral neuropathy, and conjunctivitis.	Kundu et al., 2011
3.	Taiwan; 981.	Drinking water (urine)	Thr241Met	No association with urothelial carcinoma.	Chiang et al., 2014
4	Taiwan; 540	Drinking water (urine)	Thr241Met	No association with renal cell carcinoma.	Hsueh et al, 2017
OGG1 (8-oxoguanine DNA glycosylase 1)					
1.	Vietnam; 100	Drinking water (urine)	Ser326Cys	Polymorphism was associated with a high urinary 8OHdG.	Fujihara et al., 2011
2.	Taiwan; 240	Drinking water (urine)	Ser326Cys	For individuals with high urinary As level, 326Cys/Cys polymorphism was associated with a high risk of hypertension.	Chen et al., 2012
3.	Taiwan; 280	Drinking water (urine)	Ser326Cys	No association with skin cancer.	Hsu et al., 2015
APE1 (apurinic/apyrimidinic endonuclease 1)					
1.	Vietnam; 100	Drinking water (urine)	Asp148Glu	148Asp/Glu polymorphism was associated with high urinary 8OHdG.	Fujihara et al., 2011

XPC (Xeroderma pigmentosum, complementation group C)

1.	Bangladesh; 940	Drinking water (water)	rs2228000	Polymorphism significantly modified arsenic induced skin lesion status.	Seow et al., 2015

p53

1.	Taiwan; 381	Drinking water (urine)	Arg72Pro rs1042522	Arg/Pro + Pro/Pro polymorphism was associated with a high risk of renal cell carcinom	Huang et al., 2011

***p21* (also known as *cyclin-dependent kinase inhibitor 1*)**

1.	Taiwan; 381	Drinking water (urine)	codon 31: rs1801270	No association with renal cell carcinoma	Huang et al., 2011

MDM2 (Mouse double minute 2 homolog)

1.	Taiwan; 381	Drinking water (urine)	T309G rs2279744	TG+GG genotype was associated with a high risk of renal cell carcinoma.	Huang et al., 2011

Immune response related genes

IL10 (Interleukin 10)

1.	West Bengal, India; 397	Drinking water (water, urine)	T3575A	TA/AA genotype is associated with high risks of skin lesions, ocular, and respiratory diseases.	Banerjee et al., 2011
2.	Bangladesh; 940	Drinking water (water)	rs3024496	Polymorphism significantly modified arsenic induced skin lesion status.	Seow et al., 2015

IL8 (Interleukin 8)

1	West Bengal, India; 397	Drinking water (water, urine)	T3575A	TA/AA genotype is associated with high risks of skin lesions, ocular, and respiratory diseases.	Banerjee et al., 2011

TNFα (Tumor Necrosis Factor alpha)

1	West Bengal, India; 397	Drinking water (water, urine)	G308A	GA/AA genotype is associated with high risks of skin lesions, ocular, and respiratory diseases.	Banerjee et al., 2011
2	Taiwan; 894	Drinking water (urine)	G308A	Variant of G308A is associated with urothelial carcinoma.	Wu et al., 2013

NALP2(also known as NLRP2, NLR family pyrin domain containing 2)					
1	West Bengal, India; 432	Drinking water (water, urine)	Ala1052Glu	Ala1052Glu polymorphism was associated with low risks of skin lesions, respiratory diseases, chromosomal aberrations.	Bhattacharjee et al., 2013b
**DNA methyl transferase genes**					
DNMT1, DNMT3A and DNMT3B (DNA methyl transferase 1, 3A and 3B)					
1.	Taiwan, 586	Drinking water (urine)	DNMT1 (rs8101626 and rs2228611) DNMT3A (rs34048824 and rs1550117) DNMT3B (rs1569686)	high urinary total arsenic level combined with the DNMT1 rs8101626 A/A + A/G genotype and the DNMT3A rs34048824 T/T + T/C genotype may increase the clear cell renal cell carcinoma risk in a dose-response manner, even for subjects who live in a low arsenic exposure area.	Yang et al., 2016
**Obesity related gene**					
Adiponectin					
1.	Taiwan, 778	Drinking water, sea food, rice (urine)	ADIPOQ rs182052 rs2241766 rs1501299 rs1063539	significant combined effects of obesity and the ADIPOQ rs182052 G/A+A/A genotype on the arsenic-related risk of Renal Cell Carcinoma in a population with low arsenic exposure.	Hsueh et al., 2018
**Gene related to cell signalling**					
INPP5A (Inositol Polyphosphate 5-Phophatase A)					
1.	Bangladesh; 940	Drinking water (water)	rs1133400	Minor allele carriers of the skin cancer gene INPP5A was associated arsenic-induced skin lesions in both main and replicative populations. Genetic variation in INPP5A appears to have a role in susceptibility to arsenic toxicity.	Seow et al., 2015

[As – Arsenic, iAs – Inorganic Arsenic, MMA – Monomethylated Arsenic species, DMA –Dimethylated Arsenic species]

References

Agusa, T., Iwata, H., Fujihara, J., Kunito, T., Takeshita, H., Minh, T. B., Trang P., T., K., Viet, P., H., Tanabe, S. 2010. Genetic polymorphisms in glutathione S-transferase (GST) superfamily and arsenic metabolism in residents of the Red River Delta, Vietnam. Toxicology and Applied Pharmacology, 242(3), 352–362.

Andrew, A., S., Mason, R., A., Kelsey, K., T., Schned, A., R., Carmen, J., Nelson, H., H., & Karagas, M., R., 2010. DNA repair genotype interacts with arsenic exposure to increase bladder cancer risk. Toxicol Lett. 187(1), 10–14.

Balakrishnan, P., Vaidya, D., Franceschini, N., Voruganti, V., S., Gribble, M., O., Lee, E., (2017). Association of Cardiometabolic Genes with Arsenic Metabolism Biomarkers. Environmental Health Perspectives 125(1), 15–22.

Banerjee, N., Nandy, S., Kearns, J,. K., Bandyopadhyay, A., K., Das, J., K., Majumder, P., Basu, S., Banerjee, S., Sau, T., States, C., Giri, A., K., 2011. Polymorphisms in the TNF-α and IL10 gene promoters and risk of arsenic-induced skin lesions and other nondermatological health effects. Toxicological Sciences, 121(1), 132–139.

Banerjee, N., Paul, S., Sau, T. J., Das, J. K., Bandyopadhyay, A., Banerjee, S., & Giri, A. K. (2013). Epigenetic Modifications of DAPK and p16 Genes Contribute to Arsenic-Induced Skin Lesions and Nondermatological Health Effects. 135(2), 300–308.

Beebe-Dimmer, J., L., Iyer, P., T., Nriagu, J., O., Keele, G., R., Mehta, S., Meliker, J., R., Cooney, K. A., et al., 2012. Genetic variation in Glutathione S-Transferase Omega-1, Arsenic Methyltransferase and Methylene-tetrahydrofolate Reductase, arsenic exposure and bladder cancer: A case-control study. Environmental Health: A Global Access Science Source, 11(1), 1.

Bhattacharjee, P., Das, N., Chatterjee, D., Banerjee, A., Das, J,. K., Basu, S., Banerjee, S., Majumder, P., Goswami, P., Giri, A., K., 2013. Association of NALP2 polymorphism with arsenic induced skin lesions and other health effects. Mutation Research - Genetic Toxicology and Environmental Mutagenesis, 755(1), 1–5.

Chung, C., J., Pu, Y., S., Su, C., T., Chen, H., W., Huang, Y., K., Shiue, H., S., & Hsueh, Y., M., 2010. Polymorphisms in one-carbon metabolism pathway genes, urinary arsenic profile, and urothelial carcinoma. Cancer Causes and Control, 21(10), 1605–1613.

Chung, C., J., Pu, Y., S., Su, C., T., Huang, C., Y., & Hsueh, Y., M., 2011. Gene polymorphisms of glutathione S-transferase omega 1 and 2, urinary arsenic methylation profile and urothelial carcinoma. Science of the Total Environment, 409(3), 465–470. Colin, P., Koenig, P., Ouzzane, A., Berthon, N., Villers, A., Biserte, J., and Roupret, M., 2009. Environmental factors involved in carcinogenesis of urothelial cell carcinomas of the upper urinary tract. BJU international. 104, 1436-1440.

Das, N., Giri, A., Chakraborty, S., Bhattacharjee, P., 2016. Association of single nucleotide polymorphism with arsenic-induced skin lesions and genetic damage in exposed population of West Bengal, India. Mutat Res.809, 50-56.

Drobná, Z., Del Razo, L. M., García-Vargas, G. G., Sánchez-Peña, L. C., Barrera-Hernández, A., Stýblo, M., & Loomis, D., (2012). Environmental exposure to arsenic, AS3MT polymorphism and prevalence of diabetes in Mexico. Journal of exposure science & environmental epidemiology, 23(2), 151–155. doi:10.1038/jes.2012.103

Edwards, M., Hall, J., Gong, G., & O'Bryant, S. E. (2014). Arsenic exposure, AS3MT polymorphism, and neuropsychological functioning among rural dwelling adults and elders: A cross-sectional study. Environmental Health: A Global Access Science Source, 13(1), 1–7.

Engström, K.S., Vahter, M., Fletcher, T., Leonardi, G., Goessler, W., Gurzau, E., Koppova, K., Rudnai, P., Kumar, R. and Broberg, K., 2015. Genetic variation in arsenic (+ 3 oxidation state) methyltransferase (AS3MT), arsenic metabolism and risk of basal cell carcinoma in a E uropean population. Environmental and molecular mutagenesis, 56(1), pp.60-69.

Fu, S., Wu, J., Li, Y., Liu, Y., Gao, Y., Yao, F. et al., 2014. Urinary arsenic metabolism in a Western Chinese population exposed to high-dose inorganic arsenic in drinking water: Influence of ethnicity and genetic polymorphisms. Toxicology and Applied Pharmacology, 274(1), 117–123.

Fujihara, J., Soejima, M., Yasuda, T., Koda, Y., Kunito, T., Iwata, H., Tanabe, S. and Takeshita, H., 2011. Polymorphic trial in oxidative damage of arsenic exposed Vietnamese. Toxicology and applied pharmacology, 256(2), pp.174-178.

Fujihara, J., Yasuda, T., Iwata, H., Tanabe, S., & Takeshita, H., 2016. Association of XRCC1 polymorphisms with arsenic methylation. Archives of Toxicology, 90(4), 1009–1012.

Gamboa-Loira, B., Cebrián, M. E., Salinas-Rodríguez, A., & López-Carrillo, L. (2017). Genetic susceptibility to breast cancer risk associated with inorganic arsenic exposure. Environmental Toxicology and Pharmacology, 56(July), 106–113.

Gao, S., Mostofa, M. G., Quamruzzaman, Q., Rahman, M., Rahman, M., Su, L., … Christiani, D. C. (2019). Gene-environment interaction and maternal arsenic methylation efficiency during pregnancy. Environment International, 125(January), 43–50.

Gong, G., O'Bryant, S., E., 2012. Low-level arsenic exposure, AS3MT gene polymorphism and cardiovascular diseases in rural Texas counties. Environ Res. 113, 52-7.

Hsieh, Y., C., Lien, L., M., Chung, W., T., Hsieh, F., Hsieh., P., F., Wu, M., M., Tseng, H., P., Chiou, H., Y., Chen, C., J., 2011. Significantly increased risk of carotid atherosclerosis with arsenic exposure and polymorphisms in arsenic metabolism genes. Environ Res. 111(6), 804-10.

Hsu, L.I., Chen, W.P., Yang, T.Y., Chen, Y.H., Lo, W.C., Wang, Y.H., Liao, Y.T., Hsueh, Y.M., Chiou, H.Y., Wu, M.M. and Chen, C.J., 2011. Genetic polymorphisms in glutathione S-transferase (GST) superfamily and risk of arsenic-induced urothelial carcinoma in residents of southwestern Taiwan. Journal of biomedical science, 18(1), p.51.

Hsu, L. I., Wu, M. M., Wang, Y. H., Lee, C. Y., Yang, T. Y., Hsiao, B. Y., & Chen, C. J., 2015. Association of environmental arsenic exposure, genetic polymorphisms of susceptible genes, and skin cancers in Taiwan. BioMed Research International, 2015.

Hsueh, Y. M., Lin, Y. C., Chen, W. J., Huang, C. Y., Shiue, H. S., Pu, Y. S., … Su, C. T., 2017. The polymorphism XRCC1 Arg194Trp and 8-hydroxydeoxyguanosine increased susceptibility to arsenic-related renal cell carcinoma. Toxicology and Applied Pharmacology, 332, 1–7.

Hsueh, Y. M., Chen, W. J., Lin, Y. C., Huang, C. Y., Shiue, H. S., Yang, S. M., … Su, C. T., 2018. Adiponectin gene polymorphisms and obesity increase the susceptibility to arsenic-related renal cell carcinoma. Toxicology and Applied Pharmacology, 350(250), 11–20.

Huang, C. Y., Lin, Y. C., Shiue, H. S., Chen, W. J., Su, C. T., Pu, Y. S., Hsueh, Y. M., et al., 2018. Comparison of arsenic methylation capacity and polymorphisms of arsenic methylation genes between bladder cancer and upper tract urothelial carcinoma. Toxicology Letters, 295, 64–73.

Huang, C. Y., Su, C. T., Chu, J. S., Huang, S. P., Pu, Y. S., Yang, H. Y., Hsueh, Y. M., 2011. The polymorphisms of P53 codon 72 and MDM2 SNP309 and renal cell carcinoma risk in a low arsenic exposure area. Toxicology and Applied Pharmacology, 257(3), 349–355.

Huang, C.Y., Chung, C.J., Pu, Y., S., Lin, Y., C., Wu, C., C., Shiue, H., S., Huang, Y., K., 2013. Polymorphism of inflammatory genes and arsenic methylation capacity are associated with urothelial carcinoma. Toxicology and Applied Pharmacology, 272(1), 30–36.

Huang, C.Y., Huang, Y.L., Hsueh, Y.M., Lin, Y.C., Chiang, C.I., Chen, W.J., … Shiue, H.S., 2014. XRCC1 Arg194Trp and Arg399Gln polymorphisms and arsenic methylation capacity are associated with urothelial carcinoma. Toxicology and Applied Pharmacology, 279(3), 373–379.

Hwang, Y. H., Chen, Y. H., Su, Y. N., Hsu, C. C., Chen, Y. H., & Yuan, T. H. (2010). Genetic polymorphism of As3MT and delayed urinary DMA excretion after organic arsenic intake from oyster ingestion. Journal of Environmental Monitoring, 12(6), 1247–1254.

Kaya-Akyüzlü, D., Kayaalti, Z., & Söylemezoğlu, T. (2016). Influence of MRP1 G1666A and GSTP1 Ile105Val genetic variants on the urinary and blood arsenic levels of Turkish smelter workers. Environmental Toxicology and Pharmacology, 43, 68–73.

Lesseur, C., Gilbert-Diamond, D., Andrew, A.S., Ekstrom, R.M., Li, Z., Kelsey, K.T., Marsit, C.J. and Karagas, M.R., 2012. A case-control study of polymorphisms in xenobiotic and arsenic metabolism genes and arsenic-related bladder cancer in New Hampshire. Toxicology letters, 210(1), pp.100-106.

Lin, G. F., Du, H., Chen, J. G., Lu, H. C., Guo, W. C., Golka, K., & Shen, J. H., 2010. Association of XPD/ERCC2 G 23591 A and A 35931 C polymorphisms with skin lesion prevalence in a multiethnic, arseniasis- hyperendemic village exposed to indoor combustion of high arsenic coal. Archives of Toxicology, 84(1), 17–24.

Luo, L., Li, Y., Gao, Y., Zhao, L., Feng, H., Wei, W., Sun, D., et al., (2018). Association between arsenic metabolism gene polymorphisms and arsenic-induced skin lesions in individuals exposed to high-dose inorganic arsenic in northwest China. Scientific Reports, 8(1), 1–12.

Niedzwiecki, M. M., Liu, X., Zhu, H., Hall, M. N., Slavkovich, V., Ilievski, V., … Gamble, M. V., 2018. Serum homocysteine, arsenic methylation, and arsenic-induced skin lesion incidence in Bangladesh: A one-carbon metabolism candidate gene study. Environment International, 113(December 2017), 133–142.

Pierce, B. L., Tong, L., Argos, M., Gao, J., Jasmine, F., Roy, S., Ahsan, H., et al., 2013. Arsenic metabolism efficiency has a causal role in arsenic toxicity: Mendelian randomization and gene-environment interaction. International Journal of Epidemiology, 42(6), 1862–1872.

Pierce, B. L., Kibriya, M. G., Tong, L., Jasmine, F., Argos, M., Roy, S., Ahsan, H. et al., (2012). Genome-wide association study identifies chromosome 10q24.32 variants associated with arsenic metabolism and toxicity phenotypes in Bangladesh. PLoS Genetics, 8(2).

Porter, K. E., Basu, A., Hubbard, A. E., Bates, M. N., Kalman, D., Rey, O., Skibola, C. F. et al., (2011). Association of Genetic Variation in Cystathionine-β-Synthase and Arsenic Metabolism. Environ Res . 110(6), 580–587.

Rodrigues, E. G., Kile, M., Hoffman, E., Quamruzzaman, Q., Rahman, M., Mahiuddin, G., Christiani, D. C., et al., 2012. GSTO and AS3MT genetic polymorphisms and differences in urinary arsenic concentrations among residents in Bangladesh. Biomarkers, 17(3), 240–247.

Steinmaus, C., Yuan, Y., Kalman, D., Rey, O., A., Skibola, C., F., Dauphine, D., Basu, A., Porter, K., E., Hubbard, A., Bates, M., N., Smith, M., T., and Smith. A., H., 2010. Individual Differences in Arsenic Metabolism and Lung Cancer in a Case-Control Study in Cordoba, Argentina.ToxicolApplPharmacol. 1, 247(2).

Wu, M. M., Chiou, H. Y., Chen, C. L., Hsu, L. I., Lien, L. M., Wang, C. H., Chen, C. J., et al., 2011. Association of heme oxygenase-1 GT-repeat polymorphism with blood pressure phenotypes and its relevance to future cardiovascular mortality risk: An observation based on arsenic-exposed individuals. Atherosclerosis, 219(2), 704–708.

Wu, M. M., Chiou, H. Y., Lee, T. C., Chen, C. L., Hsu, L. I., Wang, Y. H., … Chen, C. J., 2010. GT-repeat polymorphism in the heme oxygenase-1 gene promoter and the risk of carotid atherosclerosis related to arsenic exposure. Journal of Biomedical Science, 17(1), 1–11.

Yang, J., Yan, L., Zhang, M., Wang, Y., Wang, C., & Xiang, Q. (2015). Associations between the polymorphisms of GSTT1, GSTM1 and methylation of arsenic in the residents exposed to low-level arsenic in drinking water in China. Journal of Human Genetics, 60(7), 387–394.

Yang, S. M., Huang, C. Y., Shiue, H. S., Pu, Y. S., Hsieh, Y. H., Chen, W. J., … Hsueh, Y. M., 2016. Combined effects of DNA methyltransferase 1 and 3A polymorphisms and urinary total arsenic levels on the risk for clear cell renal cell carcinoma. Toxicology and Applied Pharmacology, 305, 103–110.

Yu, Y., Guo, Y., Zhang, J., Xie, J., Zhu, Y., Yan, J., Wang, B. and Li, Z., 2017. A perspective of chronic low exposure of arsenic on non-working women: Risk of hypertension. Science of The Total Environment, 580, pp.69-73.
